# Proteomic profiling of neutrophils and plasma in community-acquired pneumonia reveals crucial proteins in diverse biological pathways linked to clinical outcome

**DOI:** 10.3389/fimmu.2024.1470383

**Published:** 2024-10-18

**Authors:** Erik H. A. Michels, Osoul Chouchane, Justin de Brabander, Alex F. de Vos, Daniël R. Faber, Renée A. Douma, Eva R. Smit, W. Joost Wiersinga, Maartje van den Biggelaar, Tom van der Poll, Arie J. Hoogendijk

**Affiliations:** ^1^ Center for Experimental and Molecular Medicine (CEMM), Amsterdam UMC location University of Amsterdam, Amsterdam, Netherlands; ^2^ Department of Internal Medicine, BovenIJ Hospital, Amsterdam, Netherlands; ^3^ Department of Internal Medicine, Flevo Hospital, Almere, Netherlands; ^4^ Department of Molecular Hematology, Sanquin Research, Amsterdam, Netherlands; ^5^ Division of Infectious Diseases, Amsterdam UMC location University of Amsterdam, Amsterdam, Netherlands

**Keywords:** community-acquired pneumonia, general ward, pneumonia, proteomics, mass spectrometry, neutrophil, innate immunity

## Abstract

**Introduction:**

Neutrophils play a dichotomous role in community-acquired pneumonia (CAP), providing protection and potentially causing damage. Existing research on neutrophil function in CAP relies on animal studies, leaving a gap in patient-centered investigations.

**Methods:**

We used mass spectrometry to characterize the neutrophil proteome of moderately ill CAP patients at general ward admission and related the proteome to controls and clinical outcomes.

**Results:**

We prospectively included 57 CAP patients and 26 controls and quantified 3482 proteins in neutrophil lysates and 386 proteins in concurrently collected plasma. The extensively studied granule-related proteins in animal models did not drive the neutrophil proteome changes associated with human CAP. Proteome alterations were primarily characterized by an increased abundance of proteins related to (aerobic) metabolic activity and (m)RNA translation/processing, concurrent with a diminished presence of cytoskeletal organization-related proteins (all pathways p<0.001). Higher and lower abundances of specific proteins, primarily constituents of these pathways, were associated with prolonged time to clinical stability in CAP. Moreover, we identified a pronounced presence of platelet-related proteins in neutrophil lysates of particularly viral CAP patients, suggesting the existence of neutrophil-platelet complexes in non-critically ill CAP patients. Of the proteins measured in neutrophils, 4.3% were detected in plasma.

**Discussion:**

Our study presents new perspectives on the neutrophil proteome associated with CAP, laying the groundwork for forthcoming patient-centred investigations. Our results could pave the way for targeted strategies to fine-tune neutrophil responses, potentially improving CAP outcomes.

## Graphical Abstract

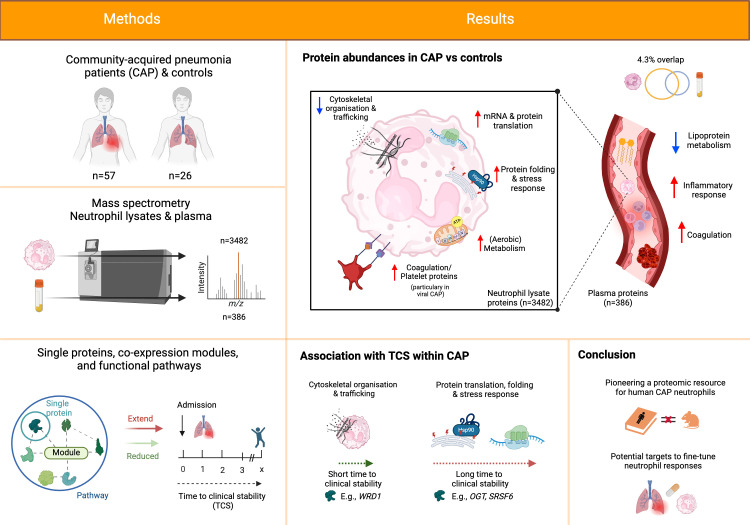

## Introduction

Community-acquired pneumonia (CAP) is a potentially life-threatening infection with substantial global health and economic burdens (1, 2). Neutrophils are the first and most abundant cellular responders to invading respiratory pathogens (3, 4). In their arsenal against pathogens, neutrophils exhibit many functions, including phagocytosis, neutrophil extracellular trap formation, and the release of reactive oxygen species (ROS), antimicrobial peptides, and serine proteases (4, 5). This diverse repertoire strongly contributes to host defense mechanisms, as exemplified by the higher incidence and greater pneumonia severity in individuals with neutrophil defects or neutropenia (3, 6–8). Moreover, experimental neutrophil depletion equally exacerbates pneumonia by reducing the clearance of pathogens and hampering lung repair after infection (9–11). While the latter highlights that neutrophils contribute to tissue repair following infections (11, 12), neutrophils can also cause harm (4). Dysregulation of apoptosis, hyperreactivity, and a disproportionate influx of neutrophils are all associated with increased lung injury in pneumonia (4, 8, 13).

Recognizing the vital role of neutrophils and the delicate balance between their beneficial and detrimental effects, researchers have advocated for the modulation of neutrophil responses in respiratory diseases and (lung) infections (14–17). Thus far, studies of neutrophils in CAP almost exclusively involved mice and *ex-vivo* models (4, 8, 18, 19). Yet, neutrophils in mice vastly differ from those in humans, amongst others, with regard to their circulating numbers, nucleus, receptors, granule proteins, defensins, and NADPH oxidase regulation (4, 20).

To provide a human-based proteomic perspective and identify potential biomarkers or targets for immunomodulation, we performed an unbiased mass spectrometry proteomics screening of neutrophils (and concurrently collected plasma) in moderately ill general ward CAP patients and controls. To translate the proteomic findings to clinical care, we correlated proteins to clinical characteristics, including time to clinical stability (TCS).

## Materials and methods

### Study design and population

Data were derived from the ELDER-BIOME study (clinicaltrials.gov identifier NCT02928367), an observational prospective study in the Netherlands. The study was approved by the medical ethical committee of the Amsterdam University Medical Center and has been previously described in detail (21, 22). In short, trained physicians screened the general ward of two peripheral hospitals and two sites of an academic hospital for patients with a high clinical suspicion of CAP. To meet the inclusion criteria, patients had to present with at least one respiratory symptom (new cough or sputum production, chest pain, dyspnea, tachypnea, abnormal lung examination, or respiratory failure) and one systemic symptom (documented fever or hypothermia, leucocytosis or leukopenia), and an evident new or progressive infiltrate, consolidation or pleural effusion on chest X-ray or computed tomography scan (21, 22). Controls were hospital employees or non-infectious patients recruited at the outpatient clinics. To characterize the severity of disease on admission, we used the MEWS (23), CURB-65 (24) and qSOFA (25) scores. As the main clinical outcome measure for CAP patients, we determined the time to clinical stability (TCS), which was defined using Halm’s criteria: temperature ≤37·2°C, heart rate ≤100 bpm, systolic blood pressure ≥90 mmHg, respiratory rate ≤24 bpm, and oxygen saturation ≥90% for the entire day (26). TCS reflects the time a patient needed to regain clinical stability or was discharged. All participants provided written informed consent. For the current analysis, patients with COVID-19 were excluded.

### Sample preparation

EDTA plasma (for plasma proteomics) and heparin blood were obtained from CAP patients within 24 hours of hospital admission. For neutrophil isolation, freshly collected blood from heparin tubes was 1:1 diluted with PBS and layered over Ficoll. Neutrophils were isolated from the lowest fraction of the Ficoll-Paque separated heparin tubes. Erythrocytes were lysed with erythrocyte lysis buffer (Sigma-Aldrich) (21). The neutrophil isolates were centrifuged and washed an additional 3 times with cold PBS, snap-frozen in liquid nitrogen, and then directly stored at -80°C. Neutrophil isolation methods were consistent with our previous publication (21), where we observed a purity of 94.1% [IQR, 86.0–97.3] using flow cytometry (FACS Canto II with FACSDiva Software; BD Biosciences) confirmed with anti-CD14-APC, anti-CD3-PECy7, and anti-CD66-FITC antibodies (BD Biosciences).

### Mass spectrometry analysis

For details, see **Supplementary Methods**. In brief, neutrophils were lysed, denatured, and sonicated. Plasma samples were diluted and alkylated. Both neutrophil lysate- and the plasma-proteins were digested into peptides overnight using trypsin. Next, tryptic digests were loaded onto Evotip Pure Tips (Evosep). Peptides were analyzed on an Evosep One liquid chromatography (LC) system (Evosep) coupled to an Orbitrap Fusion Lumos Tribrid mass spectrometer (Thermo Fisher Scientific, USA). Cells were analyzed using the extended 15 samples per day method, and plasma was with the 30 samples per day method (27). Both methods were optimized for run time and sample complexity (see **Supplementary Methods**). Peptides were ionized and introduced into an Orbitrap Fusion Lumos Tribrid mass spectrometer (Thermo Fisher Scientific). Data were acquired using Data Independent Acquisition. The raw mass spectrometry data files were processed with DIA-NN (version 1.8) using a generated library and Swissprot database (release 2021.22.04) (28). Raw MS and search/identification files obtained with DIA-NN have been deposited in the ProteomeXchange Consortium via the PRIDE partner repository with the dataset identifier PXD048675 (29).

Label-free quantification values of detected proteins were used for further analysis if supported by at least two distinct proteotypic precursors per sample. Moreover, only proteins quantified in 75% of samples in at least one condition were further explored. Missing values were imputed by normal distribution (width = 0.3, shift = 1.8), assuming these proteins were close to the detection limit.

### Statistical analysis

For more details, see **Supplementary Information**. Data was analyzed using R and Rstudio. Differences in protein abundance between CAP and controls were assessed using the limma package in R for differential expression analysis, including empirical Bayes moderation and Benjamini-Hochberg (BH) correction (30). We visualized overall differences in the proteome between CAP patients and controls using Principal Component Analyses (PCA) and volcano plots. To elucidate the biological significance, a Protein Set Enrichment Analysis (PSEA) and a single sample PSEA were performed (31, 32). Pathway results were clustered based on the similarity of proteins in each pathway using Ward’s clustering and the Enrichment plot R-package (33). To account for the age difference between CAP and controls, we performed a sensitivity analysis in which the limma and the pathway analyses included age as a covariate. To evaluate the impact of neutrophil maturity on the comparison between CAP and controls, we performed a variance partitioning analysis.

We employed a weighted “gene” correlation network analysis (WGCNA) to explore the relationships among proteins within our dataset (32, 33). WGCNA groups proteins into clusters, known as modules, based on strong positive correlation across all samples. Such correlation implies that the proteins in a module are likely involved in a shared biological pathway (34). A unique color distinctly identifies each module. For each patient, we compute a module score or module eigenprotein per module. The module score indicates the cumulative abundance of a module’s proteins within a patient: the higher the score, the more abundant the proteins of that module in that patient. Notably, elevated module scores in a particular patient subgroup (e.g., CAP compared to controls) highlight a greater presence of the module’s proteins in those individuals. To account for the age difference when comparing module scores between CAP and controls, we performed a sensitivity analysis in which age was added as a covariate. Moreover, when a clinical feature of CAP is positively correlated with a specific module score, it suggests that this clinical feature is linked with an increased abundance of the module’s proteins and vice versa (34). Unless stated otherwise, the p-values of all analyses were adjusted for multiple testing using the Benjamini-Hochberg (BH) method. The R-code of the main figures and analyses is publicly available at github.com/CEMM-NL/Proteome_Michels24.

## Results

### Cohort description

We analyzed the proteome of neutrophils and concurrently collected blood plasma in 83 subjects: 57 patients with CAP and 26 control subjects without infection recruited at the hospital (**Table 1**). For one control patient, plasma was unavailable as it was not obtained. CAP patients had a median age of 68 [58 – 76], were predominately male (56.1%), and were mainly included in the peripheral hospitals (**Supplementary Table E1**). Comorbidities mostly entailed chronic cardiac (59.6%) and respiratory disease (45.6%). In accordance with pneumonia literature^2^, no causative pathogen was identified in the majority of patients (66.7%), while Gram-positive bacteria were found in 17.5% of cases, Gram-negative bacteria in 12.3% and viruses in 7.0% of cases. Patients were moderately ill (MEWS score median [IQR]; 3 [2-4]) and often needed supplementary oxygen (84.2%). The median time for a patient to reach clinical stability (TCS) was 3 [interquartile range; 2-5] days (26). For the controls, 17 were male (65.5). Controls were slightly younger (median [IQR]; 49 [38, 61]) and suffered from comorbidities but exhibited a lower frequency of chronic respiratory diseases.

**Table 1:** Baseline characteristics and clinical course.

	CAP patients	Controls
n	57	26
Demographics		
Age, years (median [IQR])	68 [58, 76]	49 [38, 61]
Male sex, n (%)	32 (56.1)	17 (65.4)
Body mass index (median [IQR])	26.3 [23.2, 31.0]	23.8 [22.0, 25.4]
Current smoker, n (%)		
Yes	16 (28.1)	4 (15.4)
No	36 (63.2)	22 (84.6)
Unknown	5 (8.8)	0 (0.0)
Length of symptoms, days (median [IQR])	4 [3, 7]	
Comorbidities		
Charlson comorbidity index, (median [IQR])	5 [3, 6]	3 [2, 4]
Cardiac disease, n (%)	34 (59.6)	10 (38.5)
of which medicated hypertension, n (%)	23 (40.4)	4 (15.4)
Respiratory disease, n (%)	26 (45.6)	1 (3.8)
of which asthma, n (%)	9 (15.8)	1 (3.8)
of which COPD, n (%)	14 (24.6)	0 (0.0)
Diabetes, n (%)	7 (12.3)	1 (3.8)
Chronic kidney disease, n (%)	4 (7.0)	4 (15.4)
(Prior) malignancy, n (%)	5 (8.8)	0 (0.0)
Identified pathogen, n (%) 1		
Gram-positive bacteria	10 (17.5)	
Gram-negative bacteria	7 (12.3)	
Virus	4 (7.0)	
Unknown pathogen	38 (66.7)	
Disease severity on admission (median [IQR])		
qSOFA score	1 [0, 1]	
MEWS score	3 [2, 4]	
CURB-65 score	1 [1, 2]	
Routine laboratory values (median [IQR])		
Leukocyte count (x10^9^/L)	15.8 [10.7, 19.1]	
Neutrophil count (x10^9^/L)	13.0 [8.2, 16.1]	
Lymphocyte count (x10^9^/L)	0.8 [0.6, 1.2]	
Platelet count (x10^9^/L)	295 [219, 359]	
C-Reactive Protein (mg/L)	158 [73, 276]	
Creatinine (µmol/L)	86 [72, 117]	
Urea (mg/dl)	6.8 [5.1, 9.5]	
Clinical course		
Need for supplementary oxygen, n (%)	48 (84.2)	
Length of hospital stay, days (median [IQR])	4 [2, 7]	
Time to clinical stability, days (median [IQR]) 2	3 [2, 5]	
Time to clinical stability, ≤3 days, n (%)	36 (63.2)	
30-day mortality, n (%)	1 (1.8)	

COPD, chronic obstructive pulmonary disease; qSOFA quick sequential organ failure assessment; MEWS modified early warning score; CURB-65; confusion, blood urea nitrogen, respiratory rate, blood pressure, and age >65.

1 Pathogen were identified using various methods: culture (blood, sputum and pleural fluid), nasal or throat swab PCR, or urine antigen tests specific for Legionella or Streptococcus pneumoniae. Included are only those organisms cultured or identified within the first 24 hours of ward admission and subsequently confirmed as causative agents by the treating physician. Numbers do not add up to 100% as some patients suffered from multiple causative pathogens. Identified pathogens were as follows: Streptococcus pneumoniae (n=6), Influenza A virus (n=3), Haemophilus influenzae (n=3), Moraxella catarrhalis (n=2), Pseudomonas aeruginosa (n=1), Pseudomonas fluorescens (n=1), Legionella species (n=1), Respiratory Syncytial Virus (n=1), Staphylococcus aureus (n=1), Serratia marcescens (n=1), Lactobaccilus casei (n=1, from pleural fluid puncture), Streptococcus intermedius (n=1), Streptococcus milleri (n=1), Actinomyces neuii (n=1).

2 Defined as the Halm’s criteria: temperature ≤37.2°C, heart rate ≤100 bpm, systolic blood pressure ≥90 mmHg, respiratory rate ≤24 bpm, and oxygen saturation ≥90% for the entire day (26).

### Heightened metabolic and translational activities and lower cytoskeletal-related proteins in neutrophils of pneumonia patients

**Figure 1:**
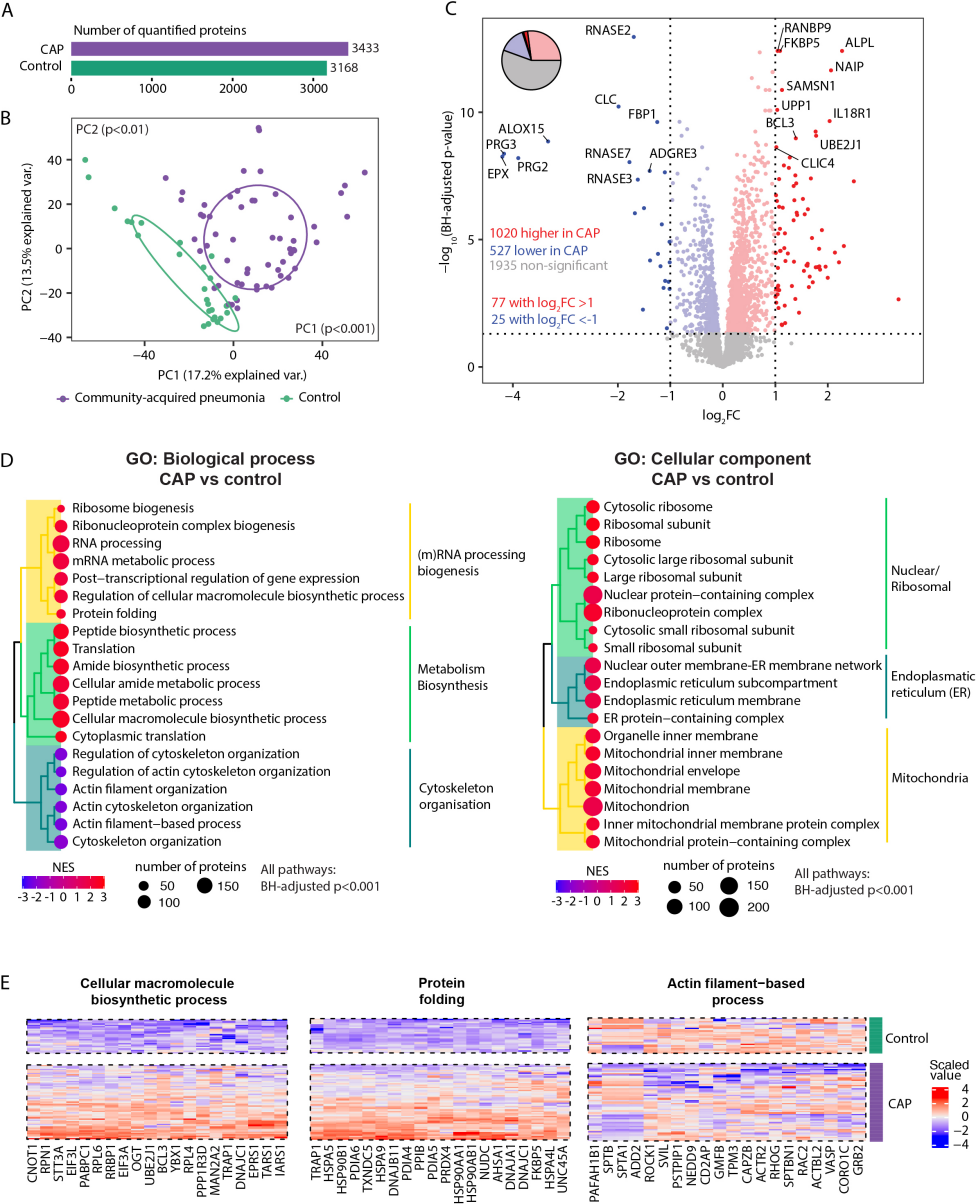
Neutrophil proteome in community-acquired pneumonia (CAP) patients and controls. **(A)** Number of quantified proteins stratified by group. **(B)** Principal component analysis (PCA) of all quantified proteins stratified by group. Each dot represents an individual subject. The ellipse indicates the central 50% of each group, color-coded as shown in the bottom part of this panel. P-values were derived from a t-test comparing the PCs between groups. **(C)** Volcano plot depicting the magnitude and significance of differences in neutrophil protein abundance between CAP patients and controls. P-values are derived from a limma differential expression analysis, including empirical Bayes moderation and Benjamini-Hochberg (BH) correction. Red dots represent proteins significantly more abundant in CAP patients, and blue dots represent proteins significantly lower in CAP patients. Dots with decreased transparency represent significant proteins with a Log_2_ fold change (FC) between -1 and 1. The pie chart visually represents the distribution of proteins with significantly different abundances between the groups. **(D)** Left panel; results of untargeted pathway analysis of the differences in neutrophil protein abundance between CAP patients and controls using the Gene ontology (GO) Biological Process database (31). The top 20 significantly different pathways are displayed and clustered based on the similarity of proteins in each pathway using Ward’s clustering and the Enrichment plot R-package (33). Normalized Enrichment Scores (NES) were used to quantify the magnitude of the difference. Right panel; similar method as left panel but now using the GO: Cellular Component database (31). **(E)** The top 20 driving proteins of the most significantly different paths per GO: biological process pathway cluster.

We identified a total of 4862 neutrophil proteins. Of these, 3433 proteins were quantified in CAP patients and 3168 in controls as they were present in at least 75% of samples per condition (**Figure 1A**). The overall neutrophil proteome differed between CAP patients and controls (both Principal Components (PCs) at least p<0.01, **Figure 1B**). Distinct patterns were detected among the total 3482 proteins quantified in either patients or controls: 1020 proteins (29.3%) exhibited higher abundances in neutrophils of CAP patients, and 527 proteins (15.1%) were lower abundant in neutrophils of CAP patients (see **Figure 1C** for the top 20 differentially expressed proteins). 1935 proteins (55.6%) were not different between patients and controls. Sheet 3 of the **Supplementary Excel File** denotes all other protein differences.

Untargeted Proteomic Set Enrichment Analysis (PSEA) revealed that neutrophils from CAP patients showed increased metabolic activity and heightened (m)RNA translation/processing, concurrent with a diminished presence of proteins linked to cytoskeletal organization (**Figure 1D** left panel, enrichment scores >0 and <0 respectively). This heightened metabolic and translational activity manifested in the cellular localization of differentially expressed proteins. Specifically, neutrophils from CAP patients exhibited notably higher abundances of ribosomal, endoplasmic reticulum, and mitochondrial proteins than neutrophils from controls (**Figure 1D**, right panel). **Figure 1E** illustrates the top 20 driving proteins of the most significantly different path per Gene Ontology (GO): Biological process pathway cluster. See sheet 4 and 5 of the **Supplementary Excel File** for a comprehensive overview of all significantly altered pathways and their proteins.

We performed several additional analyses to assess the robustness of the pathway and protein differences between CAP and controls. CAP patients were older and had a higher prevalence of COPD and asthma compared to controls. Although these variables are intrinsically connected to pneumonia development and severity (35, 36), we aimed to evaluate the influence of their imbalance on our results. Notably, correcting for these variables might reduce the observed effect of pneumonia on the proteome, as these variables may not be true confounders but rather integral components of the pneumonia condition itself. First, we replicated the primary analysis (**Figure 1**), including age as a covariate (**Supplementary Figure S1**). We observed a very high alignment of the adjusted and unadjusted results (ρ=0.98, p<0.001). In addition, the adjusted pathway analyses regarding biological processes and cellular components largely resembled the corresponding unadjusted analyses (**Figure 1D**; **Supplementary Figure S1C**, respectively). Of note, a decreased abundance of granule-related proteins in neutrophils from CAP patients ranked more often among the top 20 significantly different pathways in the adjusted analysis (top 25 in the unadjusted analysis; see sheet 5 of the **Supplementary Excel File**). Due to the absence of COPD and asthma in control subjects, adjusting for these variables was not feasible.

To determine whether the absence of chronic respiratory diseases in our control group influenced the results of our CAP – controls comparison, we used two different methods. First, we directly compared the proteomic profiles of CAP patients with COPD to those without COPD using limma, which was replicated for asthma. We observed no protein differences related to COPD and a single protein associated with asthma (see **Supplementary Excel File Sheets 9**, **10** respectively). Finally, we repeated the comparison between CAP patients and controls, this time excluding all individuals with chronic pulmonary diseases (26 CAP patients and 1 control). The exclusion of these patients had minimal impact on the neutrophil proteome comparison between the two groups, as indicated by the nearly identical volcano plot and the retention of the most discriminative proteins (**Supplementary Figure S2A**). Additionally, there was a remarkable concordance between the analysis excluding patients with respiratory comorbidities and the primary results that included all patients (ρ=0.99, p<0.001, **Supplementary Figure S2B**). Collectively, this suggests that age, COPD, and asthma do not skew the proteomic comparison between CAP patients and controls.

Given the left shift toward immature neutrophils in systemic infections, we investigated whether neutrophil maturity influenced our CAP vs. control comparison. CAP patients showed an increased abundance of immature neutrophils, as indicated by lower MME values (log2FC -1.62, adj p<0.01). To determine if neutrophil maturity drove the observed differences, we performed variance partitioning, which showed that 95.2% [IQR: 86–99%] of the protein variance remained unexplained by maturity alone. We then repeated the pathway analysis with MME as a covariate. All of the top 20 biological process pathways of the primary analysis (**Figure 1D**, left panel) remained highly significant (adj p<0.00001). Of the top cellular source pathways (**Figure 1D**, right panel), 15 out of 20 remained significant after adjustment. Pathways that lost significance were related to ribosomal structures. These results suggest that while neutrophil maturity might lead to fewer protein differences originating from ribosomal structures, the core biological processes in CAP remain unaffected.

### Decreased abundance of several ROS, granule and integrin-related proteins in pneumonia neutrophils

Adequate regulation of apoptosis, cell interactions by integrins, and release of granules, antimicrobial peptides, ROS, and serine proteases have all been described as vital components in the delicate balance between neutrophils’ beneficial and detrimental effects (4, 5, 37). Therefore, we zoomed in on essential proteins involved in these functions (**Supplementary Figure S2**) (4, 5, 37). Of the 46 detected proteins in these domains, 18 (39.1%) were lower in neutrophils of CAP patients, 6 (13.0%) were higher, and 22 (47.8%) were not different. Among the lower abundant proteins in neutrophils from CAP patients were multiple essential integrins, ROS-related, and granule-related proteins, potentially indicating their release or shedding during CAP. The proteins in neutrophils from CAP patients that had the most substantial increase were the antimicrobial peptide defensin alpha 1 (DEFA1) and the anti-apoptotic protein proliferating cell nuclear antigen (PCNA).

### Neutrophil proteomic responses in CAP compared to other bacterial infections

To explore potential CAP-specific responses, we compared our findings to the study by Kaiser et al (38), which investigated human neutrophil proteomics in an unspecified “bacterial infection” cohort. Their cohort had a smaller sample size (9 bacterial infection patients and 7 controls) with a relatively high mean SOFA score of 2.9 ± 2.2, suggesting patients with more severe disease as compared with our cohort. In the study by Kaiser et al (38), 3,179 neutrophil proteins were quantified. Of these, 2,529 overlapped with our cohort. Focusing on the 952 proteins uniquely identified in our CAP cohort (**Supplementary Figure S3**), 380 (40%) significantly differed between CAP and controls (adj p<0.05), indicating a CAP-specific neutrophil response. Among these, 253 (67%) were elevated in CAP, while 127 (33%) were reduced. Notably, key proteins such as the anti-apoptotic PCNA, NAIP (NLR Family Apoptosis Inhibitory Protein), and RNASE7 (antimicrobial peptide) were also among the most significantly altered in our primary analysis of all proteins in CAP (**Figure 1C**). On the other hand, RNASE7, SERPINB2 (anti-inflammatory), and TMOD1 (a cytoskeletal regulator) were significantly decreased in CAP compared to controls.

Pathway analysis of these CAP-specific proteins, in line with our primary analysis, revealed increased metabolic activity and protein synthesis, alongside a significant decrease in cytoskeletal organization. This increased metabolic activity potentially hints at a higher metabolic burden on neutrophils during lung infections. The downregulation of cytoskeletal organization could reflect specific functional changes or dysregulation in neutrophils during CAP. New are the pathways reflective of negative regulation of leukocyte activation. This finding indicates that despite the increased activity in terms of biosynthesis, CAP neutrophils are also being regulated to prevent excessive activation, potentially reflecting a balancing mechanism to control inflammation and avoid damaging lung tissue due to excessive neutrophil activation. Collectively, the findings highlight the distinct metabolic and functional adaptations of neutrophils in CAP, which appear tailored to the demands of fighting respiratory pathogens, further distinguishing CAP from responses observed in other bacterial infections.

### Heightened protein biosynthesis and decreased abundance of motility and cytoskeletal organization-related proteins are indicative of worse outcome in pneumonia

Next, we explored the association between the neutrophil proteome and clinical outcome. For this, we assessed the TCS, an outcome measure commonly used in studies conducted on CAP patients outside the intensive care unit (i.e., where mortality is a rare event and therefore not suitable as an outcome parameter) (26, 39–41). Noteworthy, given our uniformly ill CAP population (general ward with a MEWS of 3 [2-4]), even modest associations with TCS could be highly relevant.

**Figure 2:**
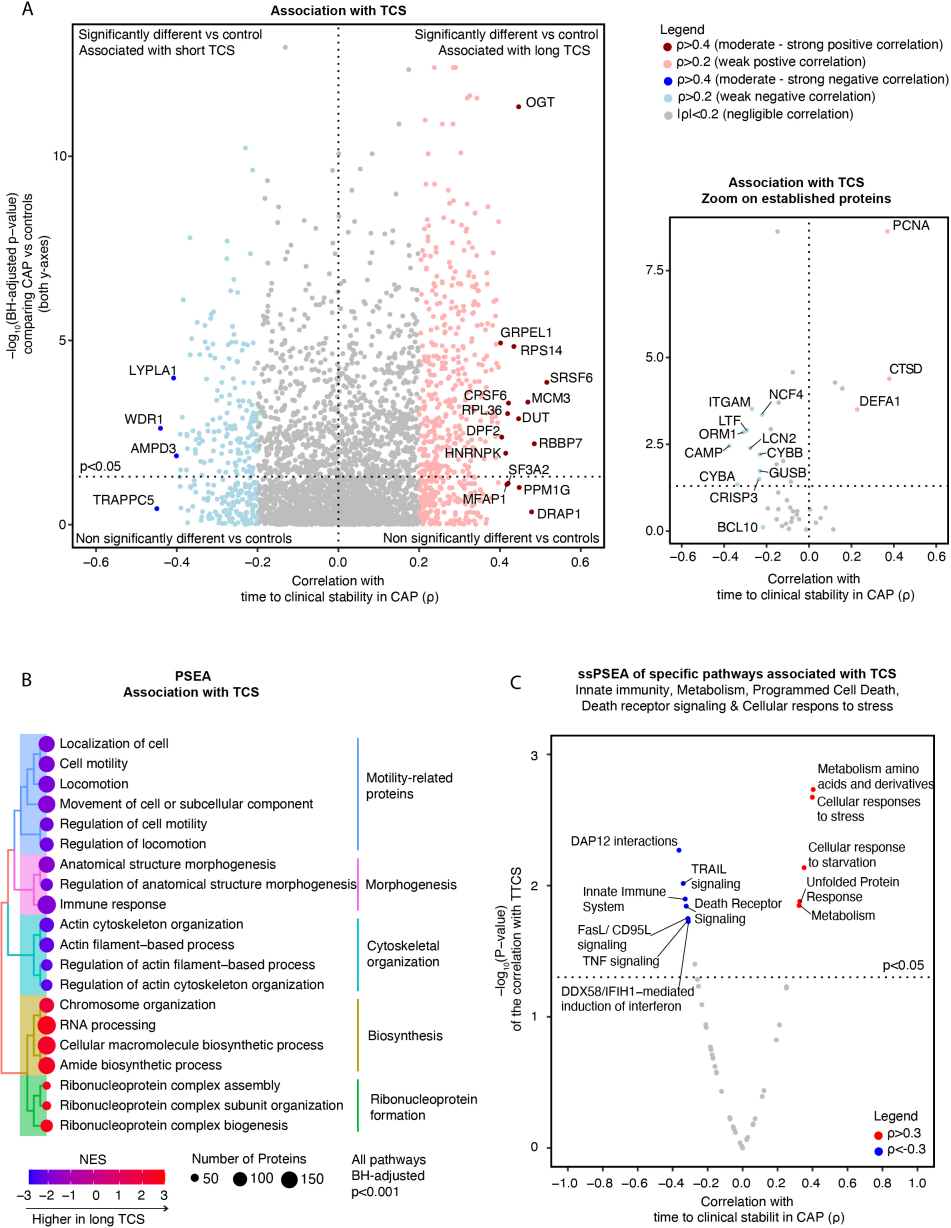
Association of neutrophil proteome in community-acquired pneumonia (CAP) patients with time to clinical stability. **(A)** Left panel; Comprehensive quadrant plot depicting the association of proteins with time to clinical stability (TCS) in CAP patients. The x-axis denotes the strength of the correlation between the protein and TCS in CAP patients derived from Spearman’s correlation test. The significance of the differences in protein abundance between CAP patients and controls was plotted on the y-axis (in line with **Figure 1C**) to facilitate interpretation. The top 20 proteins with the strongest association with TCS were labeled. Right panel: Focused Quadrant plot featuring established neutrophil proteins. This panel serves to highlight these key proteins, which are also present but unlabeled in the left panel due to their lower correlation values. **(B)** Results of an untargeted pathway analysis of proteins ranked by the strength of their Spearman’s correlation with TCS in CAP using the Gene ontology (GO) Biological Process database (31). The top 20 pathways with the strongest association with TCS were displayed and clustered based on the similarity of proteins in each pathway using Ward’s clustering and the Enrichment plot R-package (33). Normalized Enrichment Scores (NES) were used to quantify the magnitude. **(C)** Single Sample Protein Set Enrichment Analysis (ssPSEA) using the Reactome ssGSEA plugin and database in which we explored specific neutrophil-related pathways (32). The individual pathway expression values for each patient, based on the protein abundance in that patient, were correlated to TCS using a Spearman’s correlation test unadjusted for multiple testing.

A total of 826 (23.7%) proteins showed at least a weak (ρ>0.2 or ρ<-0.2) correlation with TCS, of which 527 had a positive and 299 a negative correlation (**Figure 2A**). Serine And Arginine Rich Splicing Factor (SRSF6) (ρ=0.52) and the O-linked N-acetylglucosamine transferase (OGT) (ρ=0.45) were among the proteins with the strongest association with an extended TCS. SRSF6 is a splicing factor that regulates hundreds of genes, including innate immunity, mitochondrial homeostasis, and cell death genes (42). OGT is a glycosylating enzyme and a regulator of immunometabolism as it connects the cell’s metabolic status, especially the glycemic state and the immune response (43). Conversely, WD Repeat Domain 1 (WDR1) (ρ=-0.44) and adenosine monophosphate deaminase 3 (AMPD3) (ρ=-0.40) exhibited associations with a short TCS (see sheet 12 of the **Supplementary Excel File** for a comprehensive list). WDR1 regulates neutrophil cytoskeletal remodeling (44, 45). AMPD3 catalyzes AMP deamination to inosine monophosphate (IMP) and plays a role in purine metabolism (46).

Stratifying CAP patients by the cohort’s median of TCS (≥3 days) led to similar results. SRSF6 and OGT were significantly higher in patients with prolonged TCS (≥3 days) (p=0.009 and p=0.008, respectively), while WDR1 and AMPD3 were significantly lower (p=0.009 and p=0.003) (See **Supplementary Figure S5A**). Interestingly, the associations with TCS persisted even after adjusting for clinical severity on admission (MEWS, qSOFA, and the CURB-65 score; all at least adj. p-values <0.05, except for ORM1 corrected for CURB-65 (p=0.07)).

Zooming in on the same hallmark neutrophil proteins mentioned above, the lysosomal enzyme Cathepsin D (CTSD) (ρ=0.39) and PCNA (ρ=0.37) showed the strongest positive association with TCS (**Figure 2A**), while lactoferrin (LTF) (ρ=-0.29) and orosomucoid 1 (ORM1) (ρ=-0.31) exhibited negative associations (**Supplementary Figure S4**). Similar results were observed in the stratified analysis (**Supplementary Figure S5B**), with CTSD showing a remarkable range of increase in patients with long TCS. Other integrin [e.g., Integrin alpha M (ITGAM)], apoptosis, lysosomal, granule [e.g., lipocalin 2 (LCN2 or NGAL)], ROS [e.g., Neutrophil Cytosolic Factor 4 (NCF4)] and antimicrobial-related proteins showed either no or a weak negative association with TCS. These findings suggest that most neutrophil proteins with the strongest association with adverse outcomes in CAP do not originate from the traditionally well-studied neutrophil functional pathways.

An untargeted PSEA revealed that proteins linked to motility, morphogenesis, and cytoskeletal organization exhibited the strongest associations with a short TCS, suggesting that these responses might improve the host response (**Figure 2B**). Proteins associated with an extended TCS were associated with ribonucleoprotein formation and protein biosynthesis. ‘Single-sample’ PSEA selective for innate immunity (encompassing ROS production, neutrophil degranulation, and antimicrobial peptides-related sub-pathways), metabolism, cellular stress, and apoptosis-related pathways showed that increased abundance of proteins involved in amino acid metabolism and cellular stress were associated with an extended TCS (**Figure 2C**). Conversely, individuals with a shorter TCS exhibited heightened protein abundances of specific innate immunity pathways and death receptor signaling.

### Specific neutrophil protein modules related to worse outcome in pneumonia

**Figure 3:**
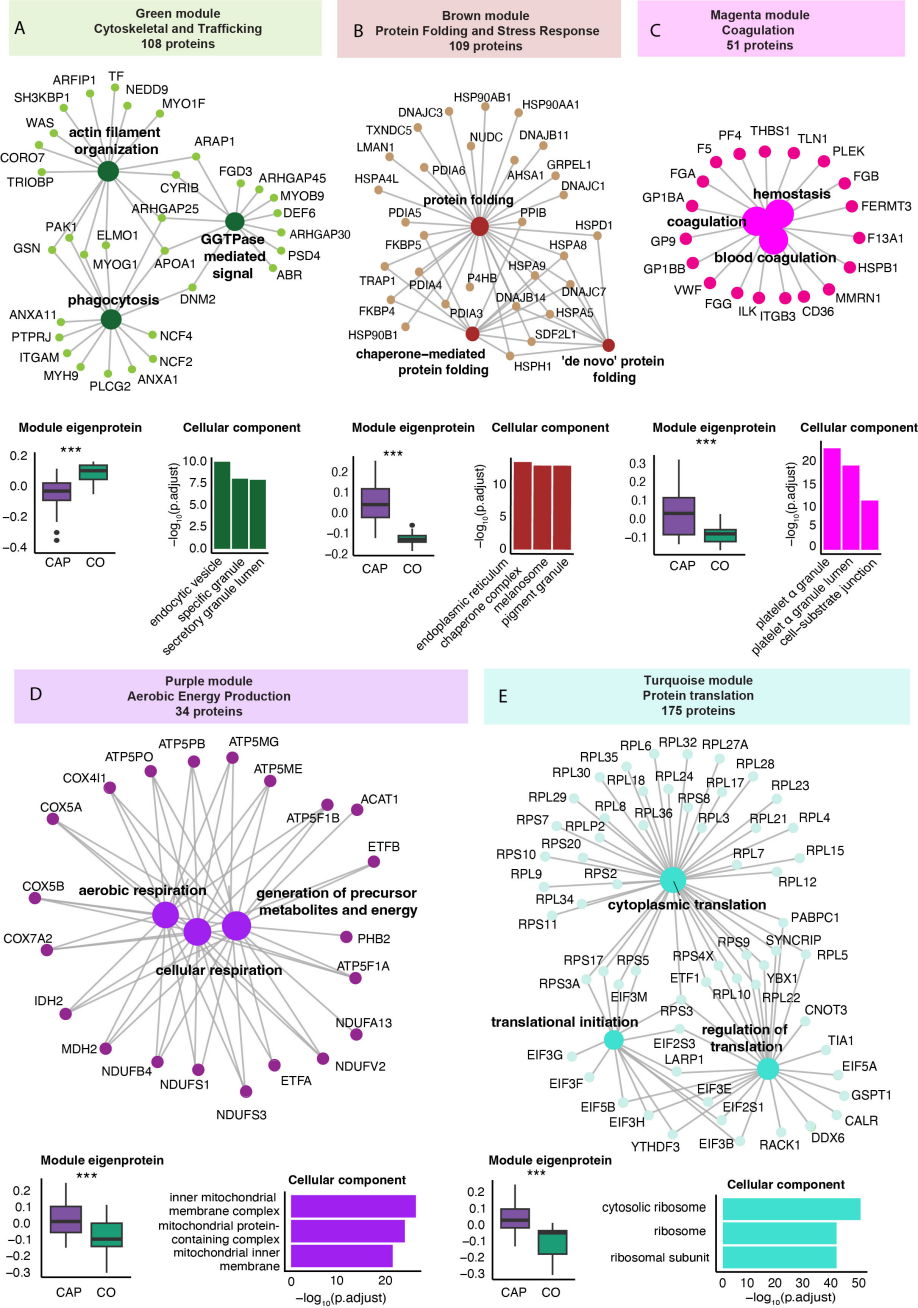
Weighted correlation network analysis of the neutrophil proteome in community-acquired pneumonia (CAP) patients and controls. **(A)** Network visualization of the three most significant Gene Ontology Biological Process (GO: BP) pathways (large dots) as determined by an overrepresentation analysis of the proteins in the green module. The numbers in the colored boxes represent the total number of proteins in the module. Displayed protein names (small dots) reflect the proteins in these top three GO: BP pathways. The boxplot in the left lower corner depicts the variation in the module score, representing the overall protein abundance pattern of all the modules’ proteins. P-values were derived from the Wilcoxon test comparing the module score between groups. The higher value of the green module score in controls (CO) compared to CAP patients indicates a higher abundance of these proteins in controls compared to CAP. The bar chart in the lower right corner reflects the three most significant GO Cellular Component pathways equally determined by an overrepresentation analysis. Collectively, this panel illustrates that CAP patients display a lower abundance of the 108 proteins in the green module (boxplot), which are functionally related to phagocytosis and cytoskeletal organization (network) and mainly originate from vesicles and granules (bar chart). **(B)** Brown protein module [methods as in **(A)**]. **(C)** Magenta protein module [methods as in **(A)**]. **(D)** Purple protein module [methods as in **(A)**]. **(E)** Turquoise protein module [methods as in **(A)**]. *** p<0.001 after Benjamini-Hochberg correction for multiple testing.

We performed a WGCNA to identify protein clusters (modules) across all samples (see methods for details). The WGCNA results in a module score per subject, which serves as an aggregate measure of the protein abundance of all proteins within that module in that individual. Five of the nine identified protein modules significantly differed between CAP patients and controls (**Supplementary Figure S6**). After removing proteins with a low module membership (KME<0.7), these modules varied from 34 to 175 proteins. Functional enrichment analysis of the differentially expressed modules using the Gene Ontology Biological Process database identified a ‘Cytoskeletal and Trafficking’ (green), ‘Protein Folding and Stress Response’ (brown), Coagulation (magenta), ‘Aerobic Energy Production’ (purple), and ‘Protein Translation’ (turquoise) module (**Figure 3**) (31). Apart from the ‘Cytoskeletal and Trafficking’ module (green), the module scores were higher in CAP patients than in controls, indicating an increased abundance of the module’s constituent proteins in CAP. Module score differences between CAP and controls were independent of age (all p<0.001).

The perhaps unexpected ‘Cytoskeletal and Trafficking’ module encompassed proteins that are directly involved in neutrophil motility [e.g., cytoskeletal proteins such as the Wiskott-Aldrich syndrome protein (WASP) and coronin 7 (CORO7)]; granule trafficking [mediated by proteins like ADP-ribosylation factor interacting protein (ARFIP1) and the SH3-domain kinase binding protein (SH3KBP1)]; and phagocytosis [which for example involves dynamin 2 (DNM2) and the NADPH oxidase complex components Neutrophil Cytosolic Factor 2 and 4 (NCF2 and NCF4)] (**Figure 3**). The ‘Protein Folding and Stress Response’ (brown) module consisted of proteins integral to the folding processes within the endoplasmic reticulum, particularly under cellular stress conditions like inflammation. This module comprises mainly Heat Shock Proteins (HSPs) and Protein Disulfide Isomerases (PDIAs), essential in preventing the accumulation of misfolded proteins that can lead to cell death and exacerbate inflammation (47).

The identification of the coagulation module, comprising key platelet-specific proteins such as platelet factor 4 (PF4), glycoprotein Ib alpha (GP1BA), and thrombospondin-1 (THBS1), indicates an enhanced formation of platelet-neutrophil complexes in patients with CAP. The presence of additional platelet-specific proteins, including multimerin 1 (MMRN1) and glycoprotein IX (GP9), within neutrophil samples from CAP patients further substantiates these interactions. Moreover, elevated levels of coagulation-related proteins such as fibrinogen alpha chain (FGA), fibrinogen beta chain (FGB), and fibrinogen gamma chain (FGG) support increased platelet activation and subsequent interaction with neutrophils.

**Figure 4:**
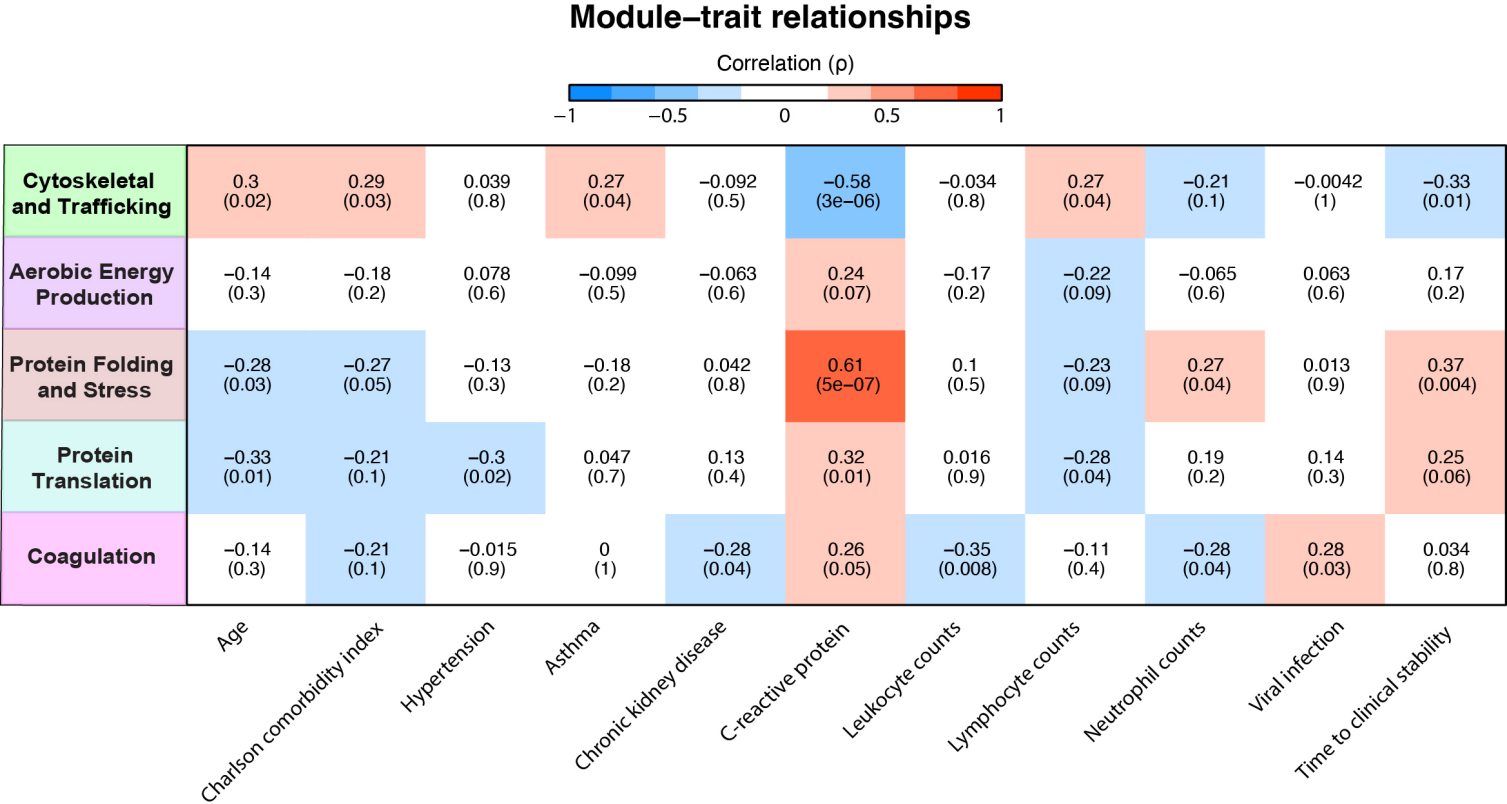
Association of neutrophil protein modules with clinical characteristics in community-acquired pneumonia (CAP) patients. Association between neutrophil protein modules and clinical characteristics in CAP patients. Only clinical characteristics with at least one significant association with a module are displayed. See sheet 12 of the **Supplementary Excel File** for all other correlations. The intensity of the red coloration denotes the strength of positive association, with deeper reds indicating stronger positive correlations. Conversely, the intensity of blue signifies the strength of negative association, with deeper blues indicating stronger negative correlations.

Subsequently, we examined the relationship between module scores and various clinical parameters within the CAP patients. A positive correlation of a variable with a specific module score indicates an increased abundance of these proteins with an increase/presence of that clinical variable or vice versa. Most notable were the ‘Cytoskeletal and Trafficking’ and the ‘Protein Folding and Stress Response’ modules, which starkly contrasted (**Figure 4**). The ‘Protein Folding and Stress Response’ module correlated with younger age, fewer comorbidities, higher CRP levels, and prolonged TCS. The green ‘Cytoskeletal and Trafficking’ module showed opposite associations. Most importantly, an increased presence of the ‘Cytoskeletal and Trafficking’ proteins was associated with a short TCS, which, therefore, may benefit the host response. Although the coagulation module contains coagulation and platelet-related proteins, its lack of a positive association with plasma platelet counts confirms that its presence may be attributed to increased neutrophil-platelet interactions rather than simply an increased number of platelets (see sheet 14 of the Excel file). Moreover, the module was associated with viral pneumonia despite those being very low in incidence (n=4, 7%). Interestingly, we observed that elevated CRP levels in plasma were strongly associated with an increase in neutrophil proteins related to cellular stress (**Figure 4**), followed by those involved in protein translation and coagulation. Although the association between CRP and proteins related to aerobic energy production was relatively modest, it remained positive. Conversely, CRP levels showed a strong inverse association with proteins related to the cytoskeleton and trafficking processes.

### Elevated inflammatory and reduced lipoprotein metabolism proteins in the plasma of pneumonia patients

**Figure 5:**
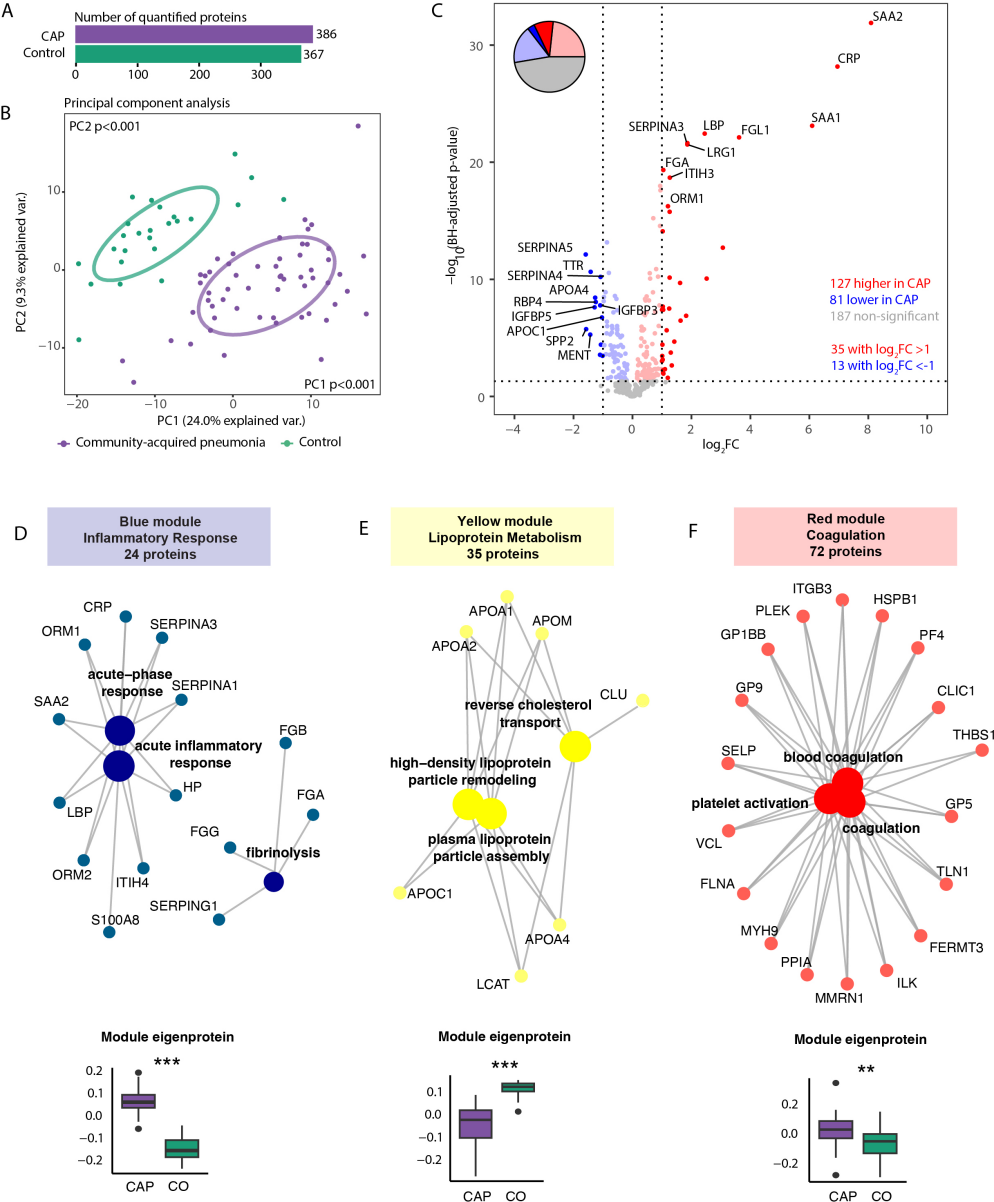
Plasma proteome in community-acquired pneumonia (CAP) patients and controls. **(A)** Number of quantified proteins stratified by group. **(B)** Principal component analysis (PCA) of all quantified proteins stratified by group. Each dot represents an individual subject. The ellipse indicates the central 50% of each group, color-coded as shown in the bottom part of the figure. P-values were derived from a t-test comparing the PCs between groups. **(C)** Volcano plot depicting the magnitude and significance of differences in plasma protein abundance between CAP patients and controls. P-values are derived from a limma differential expression analysis, including empirical Bayes moderation and Benjamini-Hochberg (BH) correction. Red dots represent proteins significantly more abundant in CAP patients, and blue dots represent proteins substantially less abundant in CAP patients. Dots with decreased transparency represent significant proteins with a Log_2_ fold change (FC) between -1 and 1. The pie chart visually represents the distribution of proteins with significantly different abundances between the groups. **(D)** Blue plasma protein module using a method similar to the neutrophil protein network analysis (see legend of **Figure 3** for an extensive explanation) **(E)** Yellow plasma protein module. **(F)** Red plasma protein module. CO: controls. **p<0.01 and ***p<0.001 after Benjamini-Hochberg correction for multiple testing.

We subsequently analyzed proteins in plasma collected concurrently with the neutrophils to assess the neutrophil proteome in the context of the circulation. We quantified 386 proteins in plasma from CAP patients and 367 in plasma from controls (**Figure 5A**). The plasma proteome differed between CAP patients and controls (both PCs at least p<0.001, **Figure 5B**). Among the 395 quantified proteins detected in the plasma of patients or controls, 127 proteins (32.2%) exhibited higher levels and 81 proteins (20.5%) lower levels in CAP patients, while 187 proteins (47.3%) did not differ between groups (see **Figure 5C** for the top 10 proteins). As expected, acute phase proteins [e.g., CRP and serum amyloid A proteins 1 and 2 (SAA1, SAA2)] were strongly increased in CAP patients (see sheet 15 of the **Supplementary Excel File** for all other proteins). Akin to the neutrophil analysis, we performed a WGCNA (**Supplementary Figure S7**). We identified three plasma protein modules significantly altered in CAP: the ‘Inflammatory Response’ (blue), ‘Coagulation’ (red), and the ‘Lipoprotein Metabolism’ (yellow) module (**Figures 5D–F**). Interestingly, the lipoprotein metabolism plasma module included several proteins with anti-inflammatory properties (e.g., APOA1, APOA4). In contrast to both other plasma modules, the proteins of the lipoprotein metabolism module were less abundant in CAP patients compared to controls. All plasma module score differences between CAP and controls were independent of age (all at least p<0.05).

### The inflammatory response in plasma is associated with profound stress, metabolic, and protein translational response in neutrophils in pneumonia

**Figure 6:**
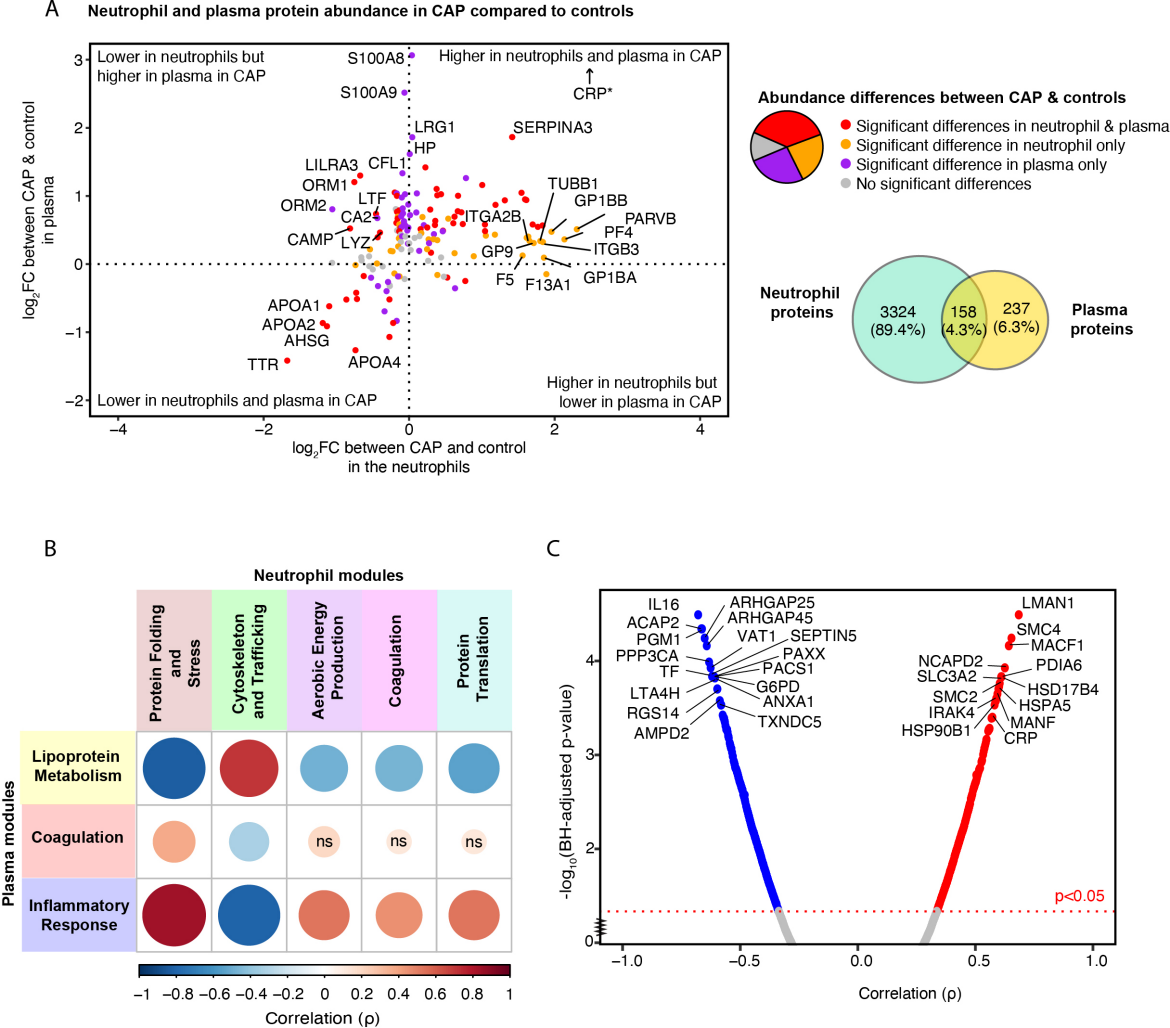
Relation between neutrophil and plasma proteome in community-acquired pneumonia (CAP) patients and controls. **(A)** Quadrant plot visualization of proteins that overlap between neutrophils and plasma. The x-axis denotes the magnitude of the difference in neutrophil protein abundance between CAP patients and controls. The y-axis indicates the difference in plasma protein abundance. Dots are colored based on the significance of comparisons. *CRP demonstrated a plasma Log_2_ fold change (FC) of 7 but was lowered to facilitate visualization. **(B)** Spearman’s correlation between neutrophil and plasma module scores. A positive correlation between modules suggests that a high abundance of the proteins comprising one module are associated with a high abundance of the proteins of the other module. Red colors represent positive associations, and blue colors negative associations. Shades of red indicate positive associations, while shades of blue denote negative associations. The magnitude of the association is depicted by both the size of the dot and the intensity of the color: larger dots and more saturated colors correspond to stronger associations. For instance, the pronounced positive correlation between the ‘Inflammatory Response’ module in plasma (blue) and the ‘Protein Folding and Stress Response’ module in neutrophils (brown) suggests that, for most patients, an increase in the plasma protein levels within the blue module corresponds with a rise in the abundance of proteins within the brown neutrophil module. **(C)** Spearman’s correlation between the inflammatory response in plasma, reflected by the blue module score, and individual neutrophil proteins in CAP patients. Abbreviations; ns: non-significant, BH; Benjamini-Hochberg.

Subsequently, we probed the correlation of proteins present in both plasma and neutrophils to discern patterns indicative of pneumonia’s systemic impact. Of the 3482 proteins detected in neutrophils, 158 (4.3%) were also detected in plasma (**Figure 6A**). Of these overlapping proteins, 58 (36.7%) were significantly different between CAP patients and controls in both compartments, 36 (22.8%) differed only in neutrophils, 40 (25.3%) only in plasma, and 24 (15.2%) in neither neutrophils nor plasma (see **Supplementary Excel File Sheets 3**, **15** for all proteins). We hypothesized that the observed increase in platelet proteins found in the neutrophils of CAP patients (heightened magenta neutrophil ‘Coagulation’ module score in CAP) could stem from enhanced formation of platelet-neutrophil complexes, as it did not correlate with plasma platelet counts. The current analysis supports the theory, showing a significant increase in proteins related to platelet and platelet adhesion (such as GP1BA, PF4, Integrin alpha-IIb (ITGA2B)) in CAP neutrophils, which is not mirrored when comparing the plasma of CAP patients to that of controls. Therefore, the heightened levels of these proteins in CAP neutrophils seem unrelated to their respective plasma protein levels.

Moreover, leukocyte immunoglobulin-like receptor 3 (LILRA3), cathelicidin antimicrobial peptide (CAMP), and ORM1 were significantly lower in neutrophils but higher in the plasma of CAP patients compared to controls, which may be due to active shedding or release of these proteins by neutrophils in CAP. A similar association was observed for the lysosome (LYZ) and lactoferrin (LTF). Proteins that showed the most substantial decrease in both neutrophils and plasma from CAP patients comprised negative acute-phase proteins, some of which possess anti-inflammatory properties (e.g., APOA1 and APOA4). This decrease in both layers could indicate consumption or decreased production.

To contextualize the non-overlapping neutrophil proteins to the plasma proteome, we determined the association between the identified neutrophil and plasma modules (**Figure 6B**). An increase in the abundance of the proteins in the plasma ‘Inflammatory Response’ module was highly correlated with an increased abundance of the proteins in the neutrophil ‘Protein Folding and Stress Response’ module, a neutrophil module associated with poorer outcomes (**Figure 4**). The ‘Inflammatory Response’ module in plasma was also associated with the ‘Aerobic Energy Production’, ‘Protein Translation’, and ‘Coagulation’ neutrophil modules, albeit to a lesser extent. On the contrary, the green ‘Cytoskeletal and Trafficking’ neutrophil module, indicative of a short TCS, was negatively associated with the ‘Inflammatory Response’ module in plasma. These results suggest that a stronger systemic inflammatory response in plasma might instigate a more profound stress, metabolic, and protein translational response in neutrophils or vice versa.

To pinpoint specific neutrophil proteins that are associated with the inflammatory response in CAP, we analyzed the correlations between individual neutrophil proteins and the ‘Inflammatory Response’ module observed in the plasma of CAP patients. 754 (21.7%) of the quantified neutrophil proteins were significantly associated with the ‘Inflammatory Response’ module in plasma (**Figure 6C**). Sheet 16 **Supplementary Excel File** denotes the correlation value of all proteins. Multiple neutrophil proteins that showed a marked correlation with the plasma inflammatory response were involved in two key processes: protein folding during cellular stress (e.g., heat shock proteins and PDIA6) and chromosome condensation, stabilization, and potentially epigenetic modification [e.g., Structural Maintenance of Chromosomes 2/4 (SMC2, SMC4)] (48). Additionally, Interleukin-1 Receptor-Associated Kinase 4 (IRAK4), a pivotal mediator in the Toll-like receptor cascade (49), exhibited a comparably strong association. Among the proteins with the strongest negative association with the plasma ‘Inflammatory Response’ module was interleukin-16, a cytokine released by neutrophils during secondary necrosis (50), and the anti-inflammatory and apoptosis-related ANXA1 and Thioredoxin domain-containing protein 5 (TXNDC5) (51). In summary, our findings indicate a positive correlation between neutrophil proteins involved in stress responses and chromosomal integrity and the inflammatory response in plasma. Conversely, proteins associated with apoptosis and those exhibiting anti-inflammatory properties negatively correlate with the inflammatory response in plasma.

## Discussion

We characterized and compared 3482 proteins in neutrophil lysates from CAP patients and controls and related expression profiles to clinical characteristics and the plasma proteome. Granule-related proteins, traditionally studied in animal and *in vitro* settings (e.g., myeloperoxidase, elastase), were not the main drivers of the substantially altered neutrophil proteome in patients with CAP. Instead, changes in the neutrophil proteome in CAP were dominated by an increased abundance of proteins related to (aerobic) metabolic activity and (m)RNA translation/processing, concurrent with a diminished presence of cytoskeletal organization-related proteins. Specific proteins, primarily constituents of these pathways, were associated with worse outcomes in CAP. A protein network analysis (WGCNA) confirmed these findings. Moreover, the WGCNA revealed that neutrophil lysates from CAP patients show an increased abundance of platelet-associated proteins unrelated to plasma platelet levels.

We identified substantial differences in the neutrophil proteome between CAP patients and controls. Proteins that were more abundant in CAP are involved in (aerobic) metabolic processes, mRNA translation, and processing, potentially reflecting cellular efforts to meet the high energy and biosynthetic needs required for an effective immune response. In agreement, we previously reported on the neutrophil transcriptome and metabolome in patients with CAP (who were different from those included in the current investigation), showing that the proinflammatory phenotype of neutrophils in CAP is associated with a high intracellular energy state (21). Interestingly, despite the need for such a shift, an increased abundance of proteins related to ribonucleoprotein complex formation, metabolism (particularly amino acids), and cellular stress in CAP was associated with a prolonged TCS in both the pathway and protein network analysis. Such a correlation might signal either increased disease severity or a state of neutrophil hyperreactivity; animal studies and *in vitro* data suggest that the latter could contribute to adverse clinical outcomes (4, 8, 13).

Our study identified OGT as a constituent of the heightened metabolic and biosynthetic pathways in neutrophils from CAP patients. OGT, a protein responsible for serine/threonine post-translational glycosylation, has emerged as a critical player in immunometabolism in experimental settings (43, 52). Previous *in vitro* studies have demonstrated that neutrophils exposed to a chemotactic peptide (fMLP) demonstrated strong OGT activity, characterized by rapidly increased levels of O-GlcNAc-modified proteins (43). Moreover, these increased O-GlcNAc-modified proteins enhanced the neutrophils’ chemotaxis and migration capacity (43). Conversely, myeloid cell-specific deletion of OGT in murine sepsis models precipitated heightened mortality and inflammation, implying a protective role for myeloid cell OGT in sepsis (52). In our study, the increased abundance of OGT in neutrophils of CAP patients correlated with a longer TCS. Taken together, these data suggest that OGT might be a candidate worth further exploring in mediating the delicate balance between neutrophils’ beneficial and detrimental effects.

SRSF6 is one of the proteins that comprised the enhanced mRNA translation and processing pathways and was substantially increased in neutrophils of CAP patients. Moreover, SRSF6 was among the proteins with the strongest association with a prolonged TCS. SRSF6 is a splicing factor that regulates hundreds of genes, including innate immunity, mitochondrial homeostasis, and cell death genes (42). In a recent study, SRSF6 was found to mediate pleural fibrosis *in vitro*, and inhibition of SRSF6 in a pleural fibrosis mouse model ameliorated lung damage (53). To our knowledge, a possible role for SRSF6 in pneumonia has yet to be examined thus far.

Proteins exhibiting lower abundance in neutrophils from CAP patients than controls were predominantly implicated in cytoskeletal organization, motility, and trafficking. Correspondingly, the ‘Cytoskeletal and Trafficking’ protein network was the only module with decreased abundance in neutrophils from CAP patients; this neutrophil module had an inverse correlation with the ‘Inflammatory Response’ module in plasma. Moreover, diminished levels of proteins in this neutrophil module correlated with poorer clinical outcomes. In fact, our untargeted pathway analysis revealed that more than half of the top 25 pathways significantly associated with TCS were related to cytoskeletal organization and motility. This association with worse outcomes in CAP might arise from the impairment of essential neutrophil functions involving cytoskeletal proteins, such as chemotaxis, phagocytosis, adhesion, and the regulation of granule and vesicle trafficking (45).

WDR1, a cytoskeletal pathway component, was notably reduced in neutrophils of CAP patients, correlating inversely with TCS. Interestingly, *WDR1* gene mutations affect neutrophil morphology, motility and function, causing a novel primary immunodeficiency termed the lazy leukocyte syndrome (44). Given that *WDR1* gene mutations lead to lazy leukocyte syndrome, lower WDR1 levels in neutrophils from CAP patients may impede neutrophilic responses, thereby lengthening TCS. These insights hint at the therapeutic potential of modulating cytoskeletal pathways to optimize neutrophil responses in CAP management.

AMPD3, a protein not directly involved in the most substantially altered pathways, was also lower in neutrophils from CAP and correlated inversely with TCS. AMPD3 plays a role in purine metabolism, and AMPD3 deficiency resulted in increased neutrophil infiltration, MPO levels, and lung hemorrhage in ischemia-reperfusion lung injury in mice (54), suggesting that AMPD3 may limit the inflammatory response and tissue damage in ischemic conditions.

In our protein network analysis, we found elevated levels of platelet-specific proteins in neutrophil lysates from patients with CAP unrelated to the plasma concentrations of these proteins, potentially indicating the formation of platelet-neutrophil complexes. While platelet-neutrophil complexes have been observed in sepsis, critically ill COVID-19, and experimental settings (55–57), our findings suggest they may already occur in moderately ill CAP patients in a general ward setting. Recent studies have highlighted that platelet-leukocyte interactions might be particularly present in viral infections, including influenza and COVID-19 (58, 59). Consistent with this, our results, despite the small number of viral cases, suggested an increased presence of these interactions in viral CAP.

Our findings reveal notable differences between neutrophil transcriptomic and proteomic responses in CAP (21). Previous transcriptomic analyses by our group of neutrophils from a comparable CAP population (in terms of age, sex, disease severity, and comorbidities) showed that genes involved in signal transduction, the innate immune response, and metabolism showed the strongest upregulation compared to control neutrophils, particularly for pathways related to rho GTPase, tyrosine kinase, MAPK signaling, neutrophil degranulation, Toll-like receptor cascades, and interleukin signaling (21). Metabolic changes included increased expression of glycogen, carbohydrate, and lipid metabolism genes, while amino acid metabolism was downregulated (21). These results starkly contrast with those seen on a proteome level. For instance, all substantially increased transcriptomic pathways related to signal transduction and innate immunity were non-significantly different between CAP and controls in the neutrophil proteome. Furthermore, while the transcriptomic analysis revealed a downregulation of genes involved in amino acid metabolism, our proteomic analysis demonstrated a significant upregulation of proteins in these pathways. Collectively, these differences highlight the complex regulation of neutrophil function during pneumonia, suggesting that transcriptomic data may not fully capture the post-transcriptional modifications, protein stability, or translational regulation that shape the neutrophil proteome.

Our study has strengths and limitations. We focused on moderately ill general ward patients, as critical illness often manifests with various immune dysregulations irrespective of the admission diagnosis (60). Moreover, this emphasis on ward patients might expose targets to prevent the onset of critical illness. Recent research suggests that key neutrophil granules (e.g., MPO) may follow a diurnal pattern in healthy humans. However, since all our patients and controls were sampled in the morning, any potential diurnal variation would not have impacted our results (61). We performed an in-depth analysis of many neutrophil lysates and plasma proteins and related these findings to clinical outcomes in CAP. However, due to the nature of the isolation method with a purity of 94.1% [IQR, 86.0–97.3], our neutrophil lysates may include minor fractions of other polymorphonuclear cells. We used TCS as an established outcome in patients with CAP admitted to a general ward, a population in which mortality is a rare event. We performed proteome analyses on bulk neutrophils, raising the possibility that part of our findings could be related to differences in maturation stages between patients and controls. We have comprehensively correlated our neutrophil proteomic data with the plasma proteome in paired CAP patients; nonetheless, the inclusion of cytokine data could have identified additional inflammatory mediators and provided further insights into specific signaling pathways and immune regulatory mechanisms involved in CAP.

Our study was observational, and no indefinite conclusion on causality can be drawn. While our study lacks direct ex-vivo or murine experiments, several of the proteins we identified as relevant to human CAP have been previously demonstrated to play important functional roles in sepsis and pneumonia using these models like OGT (43, 52), SRSF6 (53), WRD1 (44), AMPD3 (54). Our now publicly available data could guide future studies in exploring the therapeutic potential of these pathways in CAP, thereby providing valuable functional insights that complement our proteomic findings. Moreover, we could not document absolute protein concentrations since proteins were analyzed with unbiased mass spectrometry which quantifies proteins with relative quantification units. We focused on neutrophils in blood; while ethically challenging, future research could seek to analyze neutrophils (and progenitors) from the bone marrow and/or airways of CAP patients to obtain insight into proteome changes as cells transition between compartments. A key limitation of this study is the single time point (within 24 hours of admission) for sample collection; longitudinal data could provide insights into the progression of neutrophil responses at the proteome level.

This study provides an atlas of the neutrophil proteome in human CAP with detailed information on the differential expression of proteins in distinct biological pathways and functions and their association with clinical outcomes. Our results enhance the understanding of neutrophil pathobiology in human CAP and might serve as a proteomic resource for forthcoming investigations, including those aiming to optimize neutrophil responses.

## Data Availability

The datasets presented in this study can be found in online repositories. The names of the repository/repositories and accession number(s) can be found below: http://www.proteomexchange.org/ with the dataset identifier PXD048675.
