# Supplementary material for: Proteomic profiling of neutrophils and plasma in community-acquired pneumonia reveals crucial proteins in diverse biological pathways linked to clinical outcome
Source: Front Immunol. 2024 Oct 18;15:1470383. doi: 10.3389/fimmu.2024.1470383 (PMC11527607; doi:10.3389/fimmu.2024.1470383)
Supplement: Supplementary file 1 [file DataSheet1.docx]

# **Online supplementary information**

**Proteomic Profiling of Neutrophils and Plasma in Community-Acquired Pneumonia Reveals Crucial Proteins in Diverse Biological Pathways Linked to Clinical Outcome**

Erik H.A. Michels, Osoul Chouchane, Justin de Brabander, Alex F. de Vos, Daniël R Faber, Renée A Douma, Eva R. Smit, W. Joost Wiersinga, Maartje van den Biggelaar, Tom van der Poll,
Arie J. Hoogendijk

Table of Contents

[Online supplementary information 1](#_Toc176533665)

[Supplementary methods 3](#_Toc176533666)

[Missing data 3](#_Toc176533667)

[Time to clinical stability 3](#_Toc176533668)

[Details on Mass Spectrometry analysis 4](#_Toc176533669)

[Statistical analysis 5](#_Toc176533670)

[(Single Sample) Protein set enrichment analysis 6](#_Toc176533671)

[Weighted gene co-expression analysis (WGCNA) 7](#_Toc176533672)

[References 9](#_Toc176533673)

[Tables 10](#_Toc176533674)

[Table E1: Inclusion site of patients with community-acquired pneumonia 10](#_Toc176533675)

[Supplementary figures 11](#_Toc176533676)

[Figure S1: Neutrophil proteome in community-acquired pneumonia patients compared to controls correcting for age 12](#_Toc176533677)

[Figure S2: Neutrophil proteome in community-acquired pneumonia patients compared to controls when excluding patients with chronic respiratory conditions 13](#_Toc176533678)

[Figure S3: Neutrophil proteomic responses in CAP compared to other bacterial infections 14](#_Toc176533679)

[Figure S4: Targeted analysis of key neutrophil proteins, including proteins involved in apoptosis, the release of granules, antimicrobial peptides, reactive oxygen species (ROS), cell interactions and serine proteases 15](#_Toc176533680)

[Figure S5: Proteins with the strongest association with time to clinical stability in community-acquired pneumonia 16](#_Toc176533681)

[Figure S6: Identification of relevant neutrophil protein modules using weighted gene co-expression network analysis 17](#_Toc176533682)

[Figure S7: Identification of relevant plasma protein modules using weighted gene co-expression network analysis 18](#_Toc176533683)

## Supplementary methods

## Missing data

With regard to patients with community-acquired pneumonia (CAP), all reported baseline variables showed <5% missingness except for the neutrophil counts (7%), lymphocyte counts (7%) and body mass index (14%). Four patients (7%) showed both missing neutrophil and lymphocyte counts. We observed no statistical differences in demographics, comorbidities, routine laboratory values, causative pathogens, or clinical outcomes between patients with missing and without missing data. Therefore, we classified the missingness as missing at random. Controls had no missing data.

## Time to clinical stability

Generally, CAP patients admitted to the general ward show low mortality rates ^1^. Therefore, we used time to clinical stability (TCS) as an outcome measure. TCS was defined using Halm's criteria: temperature ≤37·2°C, heart rate ≤100 bpm, systolic blood pressure ≤90 mmHg, respiratory rate ≤24 bpm, and oxygen saturation ≥90% for the entire day ^1,2^. The variable reflects the time a patient needed to regain clinical stability or was discharged. The most unstable measurement was used when multiple daily vital signs were present. Given that vital signs are routinely taken in sick patients, we classified a missing vital sign as restored to normal. Stability could not be achieved on the day of admission.

## Details on Mass Spectrometry analysis

For mass spectrometry analysis of neutrophil proteomes, cells were lysed in 1% sodium deoxycholate (Bioworld), 10mM TCEP (Thermo Fisher Scientific), 40mM chloroacetamide (CAA) (Sigma-Aldrich), 100mM Tris-HCl pH8.0 (Gibco) ^3^. Lysates were incubated for 5 minutes at 95°C and sonicated for 10 minutes in a sonifier bath (Branson model 2510, Branson ultrasonics corporation), after which trypsin (Promega) was added in a 1:50 (w/w) protein ratio and digested overnight at room temperature ^3^. For plasma samples were first diluted in 100 mM Tris-HCl in a 1:60 (v/v) ratio, after which 9 μL of diluted plasma proteins were reduced and alkylated in 20mM TCEP and 80mM CAA in 100mM Tris-HCl pH8.0. The plasma proteins mixture was incubated for 5 minutes at 95°C, and trypsin (Promega) was added in a 1:50 (w/w) protein ratio and digested overnight at room temperature. Tryptic digests were transferred to an Evotip Pure tip (Evosep) according to manufacturer guidelines and separated on a 15cm × 150μm, 1.5μm Performance Column (EV1137 from EvoSep) with an Evosep One liquid chromatography system (Evosep). Cells were analyzed with the extended 15 samples per day method and plasma with the 30 samples per day method ^4^. Buffer A comprised 0.1% formic acid, and buffer B 0.1% formic acid in acetonitrile (Biosolve). Peptides were ionized and introduced into an Orbitrap Fusion Lumos Tribrid mass spectrometer (Thermo Fisher Scientific).

Data was acquired using Data Independent Acquisition (DIA), consisting of an MS1 scan from 390 to 1010 m/z, performed at 60K resolution (AGC target of 4×10^5^) with a maximum injection time of 100ms for plasma and 50 ms for cells. This was followed by MS2 data acquisition in centroid mode using 75 isolation windows of 8m/z. DIA spectra were acquired at 30K resolution (with an AGC target of 4×10^5^ for neutrophils and an AGC target of 1.5×10^5^ for plasma) with a maximum injection time of 54ms. HCD fragmentation was used in MS2 with a normalized collision energy of 23%. A default charge state of 3 for MS2 was used. Spectra were recorded in centroid mode.

The raw mass spectrometry data files were processed using the DIA-NN software (version 1.8) ^5^. Proteins and peptides were detected by querying the human SwissProt database (release 2021.22.04, number of entries #20395). Standard settings were combined with a generated library-based spectra search. The maximum number of variable modifications was set at 2. Protein Interference used was "Protein names (from FASTA)" and quantification strategy "Robust LC (high accuracy)". Raw MS and search/identification files obtained with DIA-NN have been deposited in the ProteomeXchange Consortium via the PRIDE partner repository with the dataset identifier PXD048675 ^6^.

Detected proteins were filtered for proteotypic peptides and ≥2 unique precursors per protein. Moreover, only proteins quantified in 75% of samples in at least one condition were further explored. LFQ values were transformed in the log_2_ scale. Missing values were imputed by normal distribution (width = 0.3, shift = 1.8), assuming these proteins were close to the detection limit.

## Statistical analysis

Histograms and quantile-quantile plots assessed data distributions. Categorical variables were analysed using a Chi-square test of independence. Non-normal continuous data were analysed using a Mann–Whitney U test, normally distributed continuous data using a student's t-test. Differences in protein abundance between CAP and controls were assessed using the limma package in R for differential expression analysis, including empirical Bayes moderation and Benjamini-Hochberg (BH) correction ^7^. The protein, pathway, and module results have been comprehensively documented in the supplementary Excel file, which might serve as a proteomic resource for future investigations.

### (Single Sample) Protein set enrichment analysis

To understand the biological significance of the differences in protein profiles between patients with CAP and controls, we conducted an unbiased Protein Set Enrichment Analysis (PSEA) using t-scored ranked proteins. When analyzing neutrophil proteins, we utilized the Gene Ontology databases for Biological Processes and Cellular Components ^8^. We exclusively employed the Gene Ontology for Biological Processes for plasma proteins, given the known source of these proteins (plasma). To facilitate the interpretation of the pathway results, we clustered the pathways based on the similarity of proteins in each pathway using Ward’s clustering and the Enrichment plot R-package ^9^.

In our quest to identify functional pathways associated with TCS in CAP, we performed Spearman's correlation tests to establish the correlation between each protein and TCS. Subsequently, we subjected these proteins, ranked by Spearman's correlation coefficient (Rho), to an unbiased PSEA using the above-described databases.

To specifically assess the relationship between hallmark neutrophil pathways in CAP and TCS, we conducted a Single Sample Protein Set Enrichment Analysis (ssPSEA) using the Reactome ssGSEA plugin and database (5). In contrast to a ‘regular’ PSEA, this approach yields individual pathway expression values for each sample based on the protein concentrations in that patient. We selected the following pathways and their related sub-pathways for this analysis: Innate Immune System (R-HSA-168249, encompassing reactive oxygen species, neutrophil degranulation, and antimicrobial peptides-related sub-pathways), Metabolism (R-HSA-1430728), Programmed Cell Death (R-HSA-5357801), Death Receptor Signalling (R-HSA-73887), and Cellular Response to Stress (R-HSA-2262752).

### Weighted gene co-expression analysis (WGCNA)

We conducted a weighted ‘gene’ co-expression network analysis (WGCNA) using the signed method ^10,11^. This method is used to discover clusters of genes or proteins that show a strong positive correlation (co-expression) ^10^ initially designed for gene data, terms related to the methods often contain the word ‘gene’ (e.g., eigengene), which in protein analyses is often replaced by ‘protein’ (e.g., eigenprotein). A step-by-step approach has been described in detail by Langfelder and Horvath (2008) ^10^. We searched for outliers using hierarchical average linkage clustering. No outliers were observed in either the neutrophils or plasma WGCNA. Next, we constructed a ‘protein’ scale-free network. The optimal power to obtain a scale-free network was based on the "pickSoftThreshold" of the WGCNA R-package, in which we had a power value ranging from 1 to 20 based on the independence and mean connectivity ^10^. The scale-free network's optimal power (β) for the neutrophils analysis was 12. For the plasma analysis, the optimal power was 6. Pearson's correlation matrix of all protein pairs was transformed into an adjacency matrix and topological overlap matrix using the obtained power value. We identified modules, which are groups of proteins with a strong positive correlation, using the dynamic tree-cut algorithm with a minimal cluster size of 40 proteins. To facilitate interpretability, we merged modules with a strong correlation (correlation >0.75) following previous publications ^11–13^.

To improve readability and minimize noise, we added an additional filtering step in analogy with recent publications ^11,13,14^. First, we calculated the eigenprotein-based connectivity (KME), a continuous measure of Module Membership for each protein per module ^10^. The KME/MM reflects the correlation of a protein with the first principal component (PC) of that module, termed the module eigenprotein. Proteins with a high KME/MM are more aligned with the module's core as they strongly associate with the module's score. For the current analysis, proteins weakly correlated to each of the modules (KME<0.7) were not assigned ^11,14^. Relevant modules were identified based on their significant differences between CAP and control groups. We then conducted a functional enrichment analysis for these modules by performing an overrepresentation analysis of the module's proteins using the Gene Ontology Biological Process database ^8^.

Additionally, to determine the cellular sources of the proteins within these modules, we carried out an overrepresentation analysis using the GeneOntology Cellular Component database ^8^. In an exploratory analysis, we evaluated the modules' clinical relevance by correlating each patient's module scores to table one variables, which included demographics, comorbidities, chronic medications, causative pathogens, disease severity on admission, routine laboratory values and TCS. Only clinical characteristics in which at least one association with a module showed a significant association were displayed in the module heatmaps to enhance readability. All other correlations were documented in the supplementary Excel file.

# References

1. Schuurman, A. R. *et al.* The host response in different aetiologies of community-acquired pneumonia. *EBioMedicine* **81**, (2022).

2. Halm, E. A. *et al.* Time to clinical stability in patients hospitalized with community-acquired  pneumonia: implications for practice guidelines. *JAMA* **279**, 1452–1457 (1998).

3. Groten, S. A. *et al.* Multi-omics delineation of cytokine-induced endothelial inflammatory states. *Commun Biol* **6**, 525 (2023).

4. Krieger, J. R. *et al.* Evosep One Enables Robust Deep Proteome Coverage Using Tandem Mass Tags while Significantly Reducing Instrument Time. *J Proteome Res* **18**, 2346–2353 (2019).

5. Demichev, V., Messner, C. B., Vernardis, S. I., Lilley, K. S. & Ralser, M. DIA-NN: neural networks and interference correction enable deep proteome coverage in high throughput. *Nat Methods* **17**, 41–44 (2020).

6. Perez-Riverol, Y. *et al.* The PRIDE database resources in 2022: a hub for mass spectrometry-based proteomics evidences. *Nucleic Acids Res* **50**, D543–D552 (2022).

7. Smyth, G. K. limma: Linear Models for Microarray Data BT  - Bioinformatics and Computational Biology Solutions Using R and Bioconductor. in (eds. Gentleman, R., Carey, V. J., Huber, W., Irizarry, R. A. & Dudoit, S.) 397–420 (Springer New York, New York, NY, 2005). doi:10.1007/0-387-29362-0_23.

8. The Gene Ontology resource: enriching a GOld mine. *Nucleic Acids Res* **49**, D325–D334 (2021).

9. Murtagh, F. & Legendre, P. Ward’s Hierarchical Agglomerative Clustering Method: Which Algorithms Implement Ward’s Criterion? *J Classif* **31**, 274–295 (2014).

10. Langfelder, P. & Horvath, S. WGCNA: an R package for weighted correlation network analysis. *BMC Bioinformatics* **9**, 559 (2008).

11. Michels, E. H. A. *et al.* Association between age and the host response in critically ill patients with sepsis. *Crit Care* **26**, 385 (2022).

12. Li, Z., Li, Y., Wang, X. & Yang, Q. PPP2R2B downregulation is associated with immune evasion and predicts poor clinical outcomes in triple-negative breast cancer. *Cancer Cell Int* **21**, 13 (2021).

13. Schuurman, A. R. *et al.* Inflammatory and glycolytic programs underpin a primed blood neutrophil state in patients with pneumonia. *iScience* **26**, (2023).

14. Lou, Y. *et al.* Characterization of transcriptional modules related to fibrosing-NAFLD progression. *Sci Rep* **7**, 4748 (2017).

# Tables

## **Table E1: Inclusion site of patients with community-acquired pneumonia**

|  |  |
| --- | --- |
| n | 57 |
| Inclusion site, n (%) |  |
| Amsterdam UMC, location AMC* | 7 (12.3) |
| Amsterdam UMC, location VUMC* | 10 (17.5) |
| BovenIJ Hospital | 11 (19.3) |
| Flevo Hospital | 29 (50.9) |

*Academic hospital

# Supplementary figures

**Figure S1: Neutrophil proteome in community-acquired pneumonia patients compared to controls correcting for age**
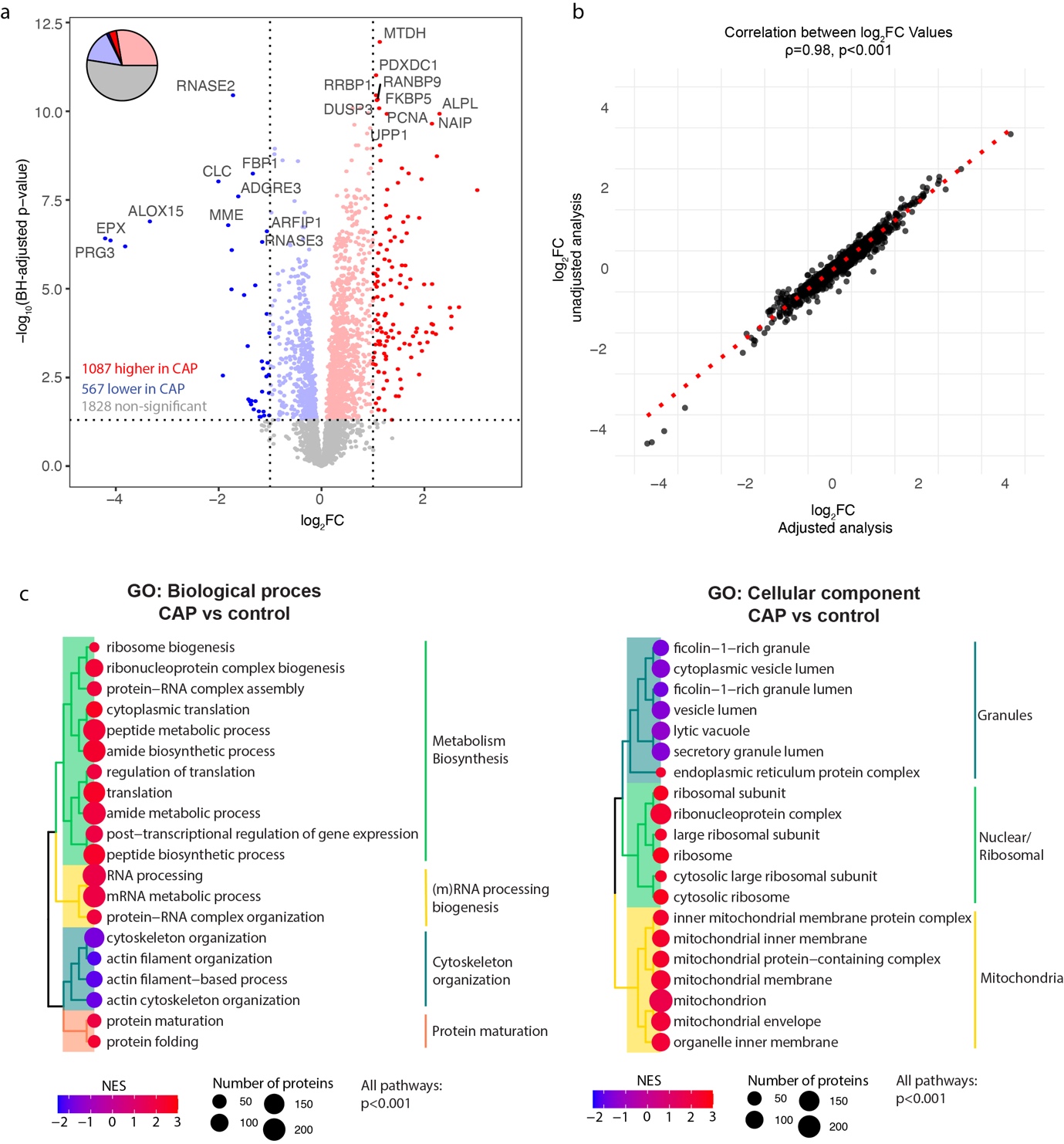


**Description Figure S1:** a) Volcano plot depicting the magnitude and significance of differences in protein abundance in neutrophils of community-acquired pneumonia (CAP) patients and controls. P-values are derived from a limma differential expression analysis, including empirical Bayes moderation and Benjamini-Hochberg (BH) correction in which age was included as a covariate. b) Spearman's correlation of the Log_2_ fold change (FC) differences in neutrophil protein abundance between CAP patients and controls in the unadjusted and the adjusted analysis c) Left panel; results of an unbiased pathway analysis of the differences in neutrophil protein abundance between CAP patients and controls using the Gene ontology (GO) Biological Process database in which age was included as a covariate ^8^. The top 20 significantly different pathways were displayed and clustered based on the similarity of proteins in each pathway using Ward's clustering and the Enrichment plot R-package ^9^. Normalized Enrichment Scores (NES) were used to quantify the magnitude of the difference. Right panel; similar method as left panel but now using the GO: Cellular Component database.

## **Figure S2: Neutrophil proteome in community-acquired pneumonia patients compared to controls when excluding patients with chronic respiratory conditions**


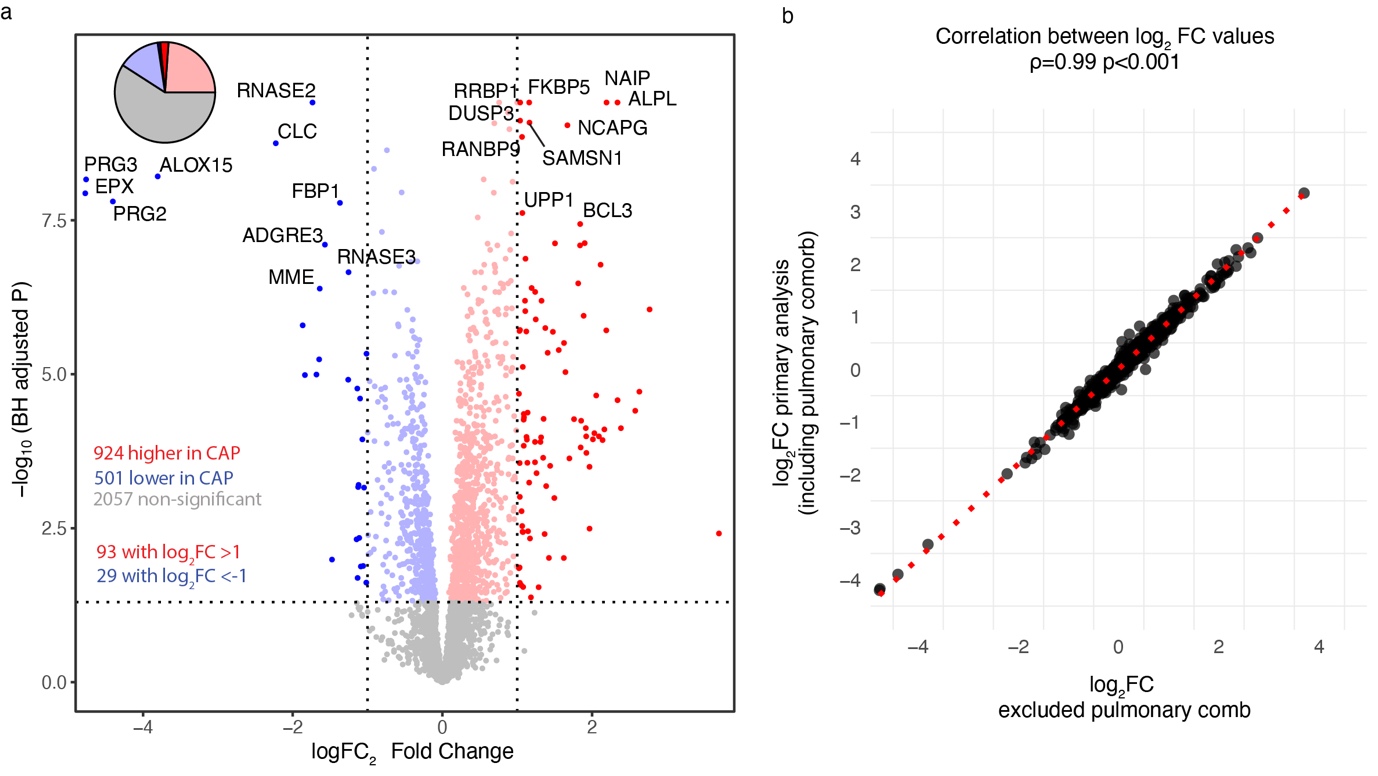

**Description Figure S2:** a) Volcano plot depicting the magnitude and significance of differences in protein abundance in neutrophils of community-acquired pneumonia (CAP) patients and controls excluding all chronic respiratory diseases. P-values are derived from a limma differential expression analysis, including empirical Bayes moderation and Benjamini-Hochberg (BH) correction. b) Spearman's correlation of the Log_2_ fold change (FC) differences in neutrophil protein abundance between CAP patients and controls in the primary analysis and the analysis in which patients with chronic respiratory infections were excluded.

## **Figure S3: Neutrophil proteomic responses in CAP compared to other bacterial infections**


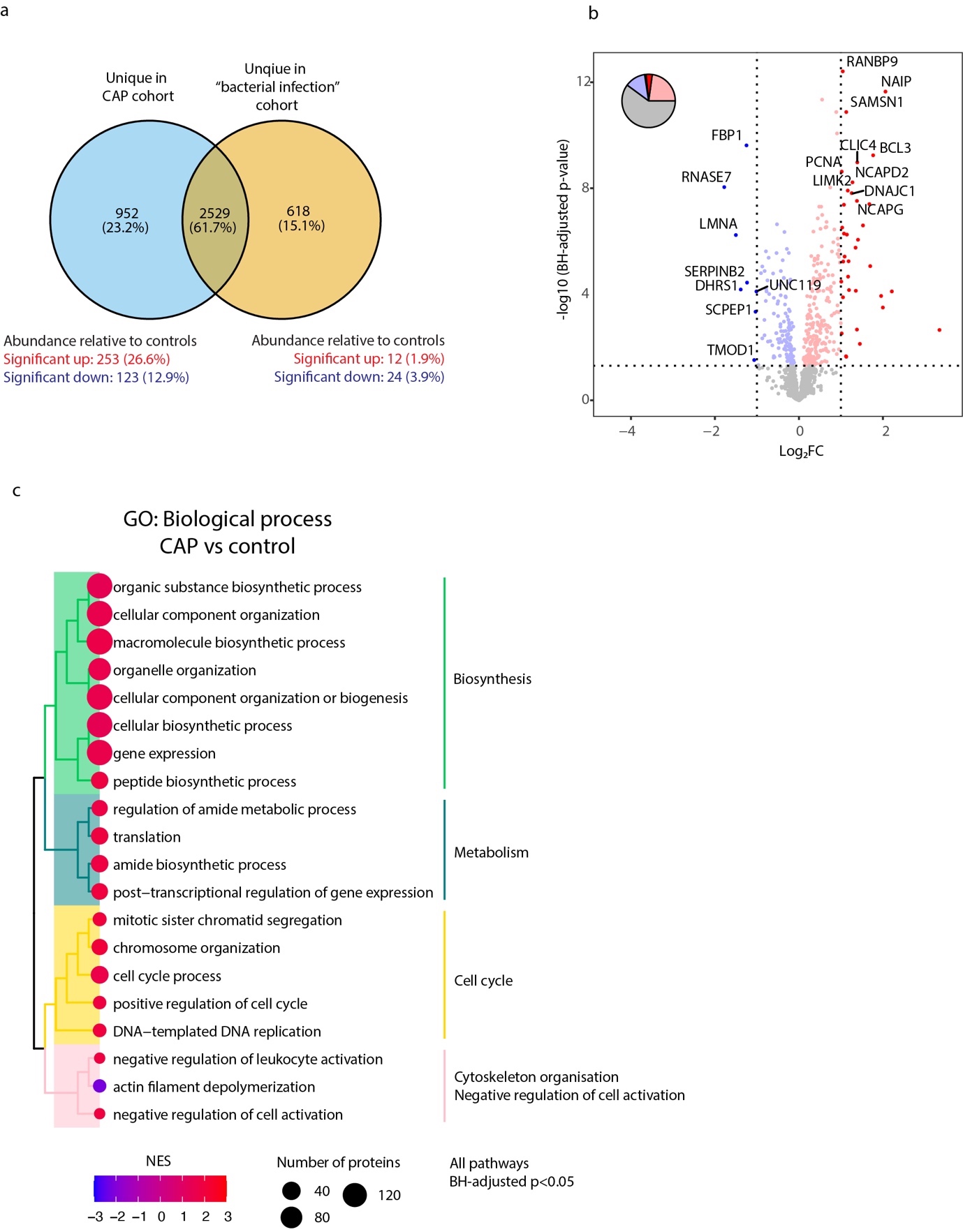


**Description Figure S3**: a) Venn diagram comparing the number of identified neutrophil proteins in both our community-acquired pneumonia (CAP) cohort and the “Bacterial infection” cohort by Kaiser et al.^10^. The numbers underneath reflect the proportion of cohort-unique proteins that significantly differed from controls in the respective cohort. B) Volcano plot of the CAP-unique proteins (n=952) depicting the magnitude and significance of the differences in the protein abundance in neutrophils of CAP patients and controls. P-values are derived from a limma differential expression analysis, including empirical Bayes moderation and Benjamini-Hochberg (BH) correction. C) Results of an untargeted pathway analysis of these differences in neutrophil protein abundance between CAP patients and controls using the Gene ontology (GO) Biological Process database incorporating only the CAP-specific proteins.

**Figure S4: Targeted analysis of key neutrophil proteins, including proteins involved in apoptosis, the release of granules, antimicrobial peptides, reactive oxygen species (ROS), cell interactions and serine proteases


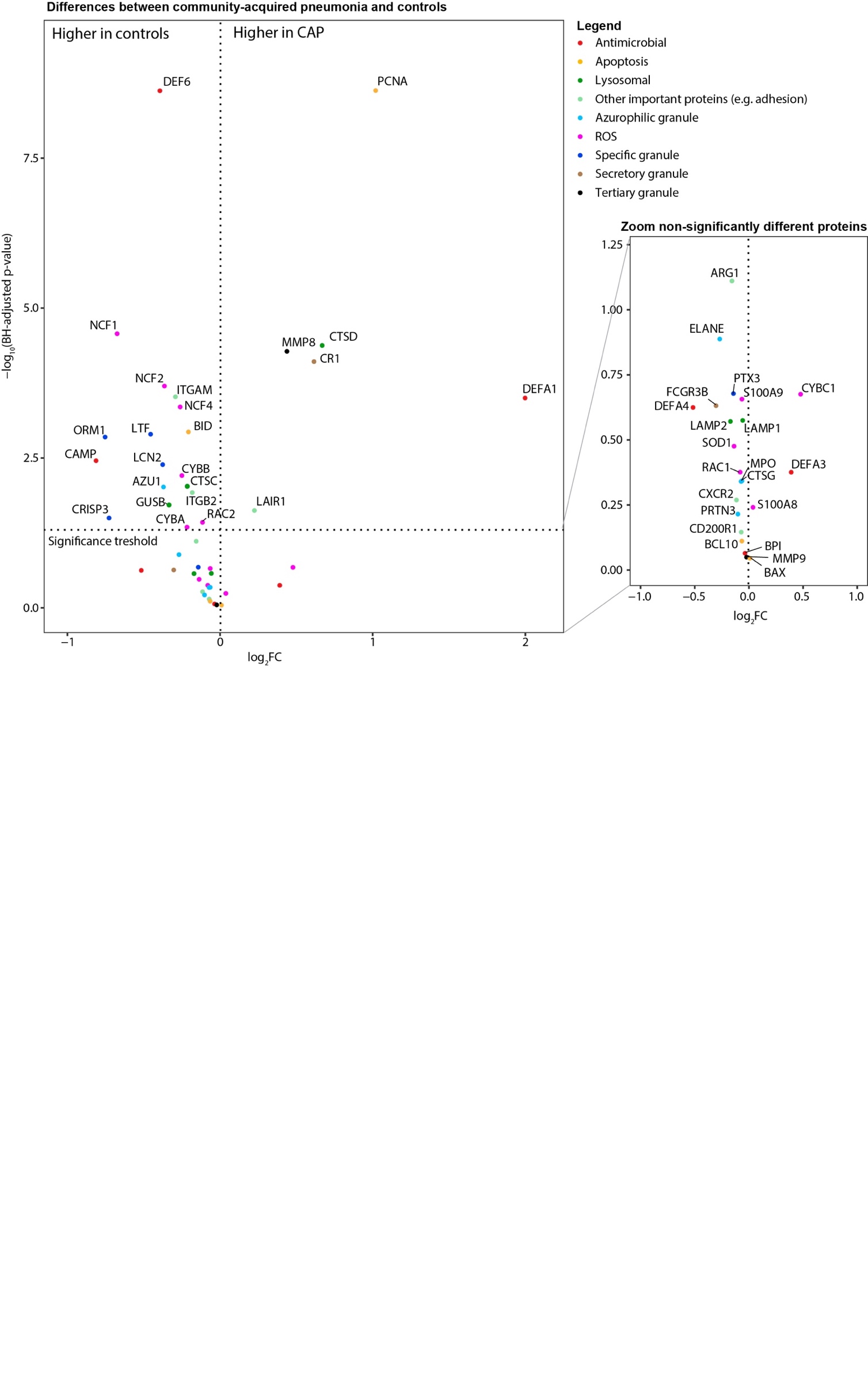

Description Figure S4:** Volcano plot depicting the magnitude and significance of differences in protein abundance in neutrophils of community-acquired pneumonia (CAP) patients and controls, zooming in on key neutrophil proteins, including proteins involved in apoptosis, release of granules, antimicrobial peptides, ROS and serine proteases. P-values were derived from the primary analysis and are concurrent with Figure 1.

## **Figure S5: Proteins with the strongest association with time to clinical stability in community-acquired pneumonia**


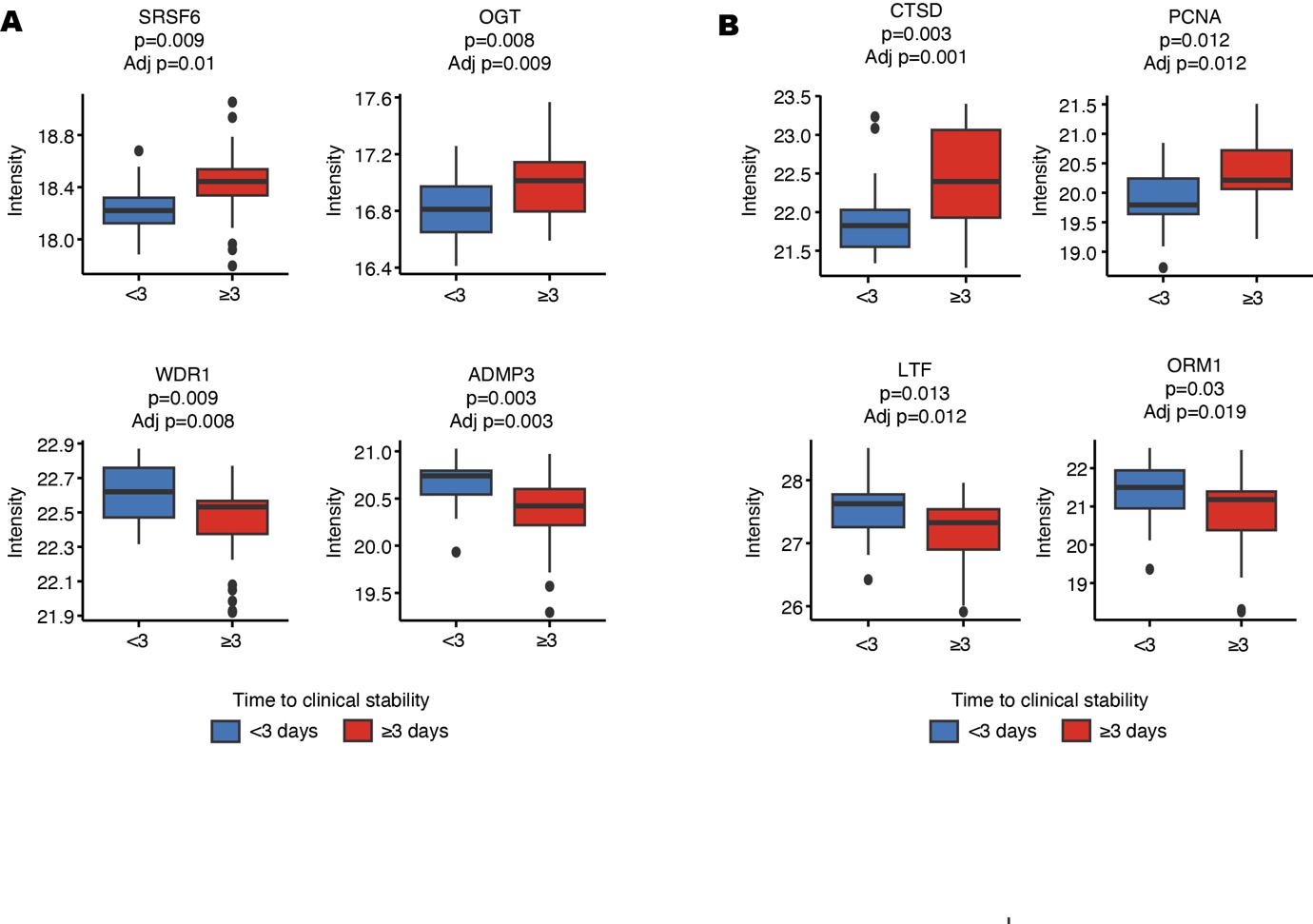


**Description Figure S5:** Box plots and whiskers representing the protein intensity stratified by the cohort’s median time to clinical stability (3 days). P-values are derived from a logistic regression. For the adjusted p-values (Adj p) the MEWS score was added as a covariate. A) The top 2 proteins with the strongest positive and negative association with time to clinical stability on a continuous scale. B) The top 2 established neutrophil proteins have the strongest positive and negative association with time to clinical stability continuously.

**Figure S6: Identification of relevant neutrophil protein modules using weighted gene co-expression network analysis
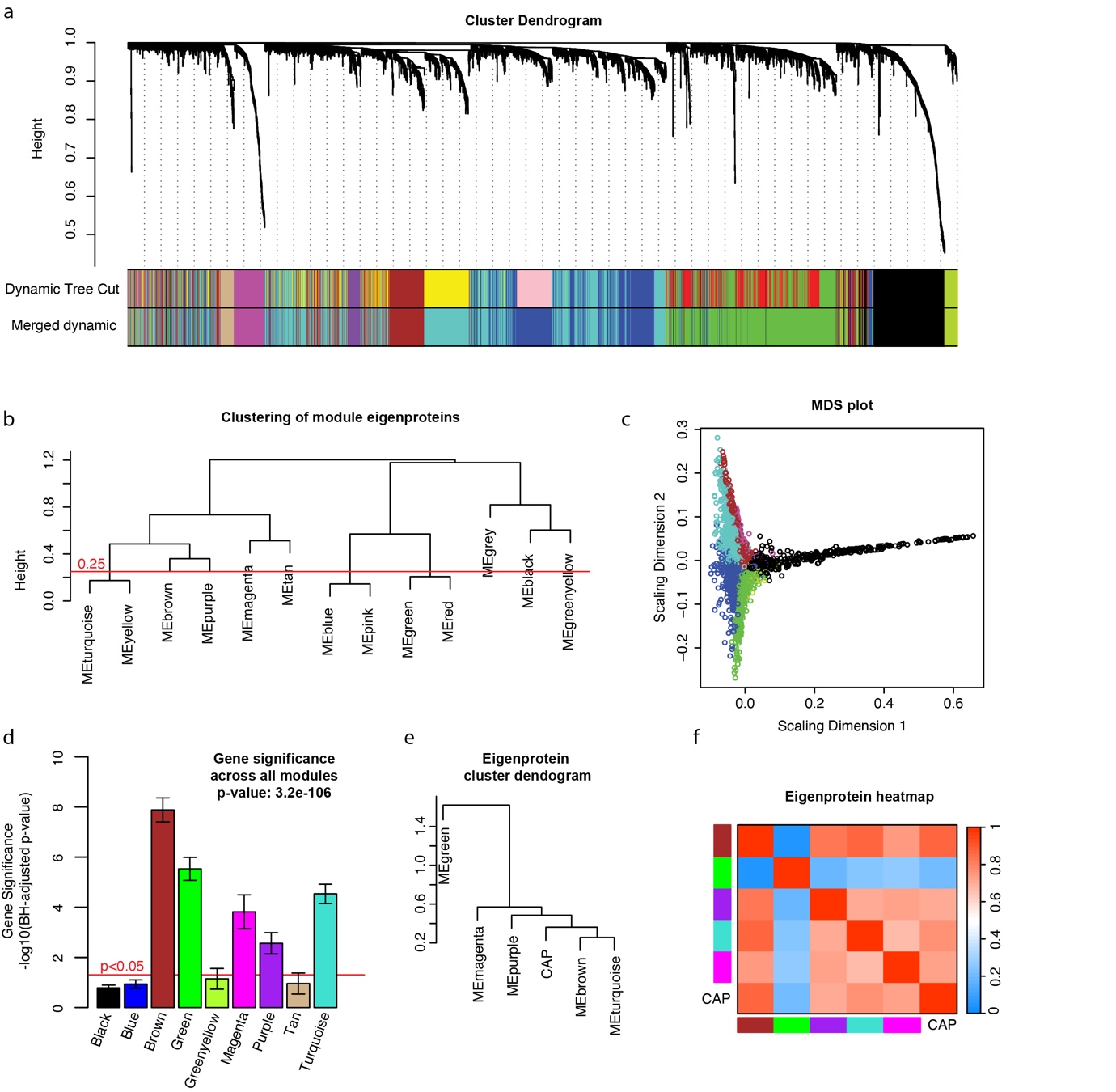
Description Figure S6:** a) Cluster dendrogram and assigned modules before and after merging similar modules. The clustering of proteins was based on dissimilarity (1 – values of the topological overlap matrix). The minimal cluster size, before the filtering of proteins with a low module membership, was set to 40 proteins. Branches characterise groups of highly correlated proteins, represented by color. The clustering of 3482 proteins of 83 patients resulted in 13 modules. b) Clustering of module eigenproteins. Modules with a correlation coefficient greater than 0.75 were merged. c) Multi-Dimensional Scaling (MDS) plot displaying the relationships between patients based on their protein expression profiles. Each point on the plot represents an individual patient. The proximity of the points reflects the similarity or dissimilarity in protein expression patterns. Patients with similar protein profiles are closer to each other, while those with dissimilar profiles are more distant. The color coding of the points is determined by the grouping of patients based on their protein expression patterns. d) Barplot of mean protein significance across modules after removing proteins with a low module membership (datKME). Higher protein significance in a module resembles a more substantial relationship of the module with community-acquired pneumonia (CAP). e) Cluster dendrogram of the significant modules, including the trait "CAP". f) Heatmap of the adjacencies in the eigenprotein network showing the module-module and module-trait (CAP) relationships. Modules are labeled by their corresponding color. Red colors indicate high adjacency (positive correlation), and blue colors show low adjacency (negative correlation).­­

## **Figure S7: Identification of relevant plasma protein modules using weighted gene co-expression network analysis**

**
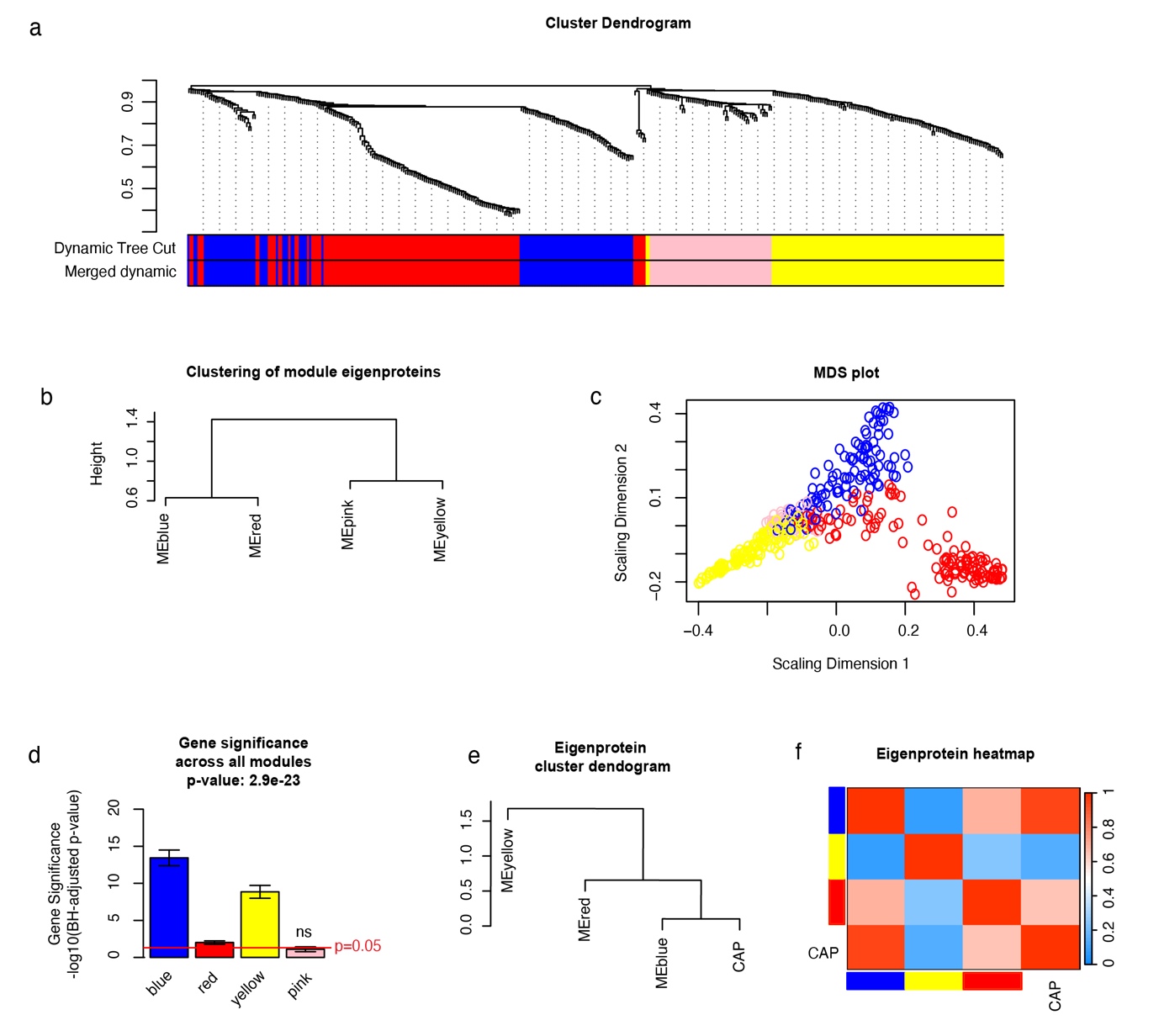
**

**Description Figure S7:** a) Cluster dendrogram and assigned modules before and after merging similar modules. The clustering of proteins was based on dissimilarity (1 – values of the topological overlap matrix). The minimal cluster size, before the filtering of proteins with a low module membership, was set to 40 proteins. Branches characterize groups of highly correlated proteins, which are represented by colors referred to as modules. The clustering of 392 proteins of 82 patients resulted in 4 modules. b) Clustering of module eigenproteins. No module showed a correlation coefficient greater than 0.75. c) Multi-Dimensional Scaling (MDS) plot displaying the relationships between patients based on their protein expression profiles. Each point on the plot represents an individual patient. The proximity of the points reflects the similarity or dissimilarity in protein expression patterns. Patients with similar protein profiles are closer to each other, while those with dissimilar profiles are more distant. The color coding of the points is determined by the grouping of patients based on their protein expression patterns. d) Barplot of mean protein significance across modules after removing proteins with a lower module membership (datKME). Higher protein significance in a module resembles a more substantial relationship of the module with community-acquired pneumonia (CAP). e) Cluster dendrogram of the significant modules, including the trait "CAP". f) Heatmap of the adjacencies in the eigenprotein network showing the module-module and module-trait (CAP) relationships. Modules are labelled by their corresponding color. Red colors indicate high adjacency (positive correlation), and blue colors show low adjacency (negative correlation)
